# Multi-voxel MR-spectroscopy signatures and associations with EEG network hyperexcitability and clinical symptomatology in borderline personality disorder

**DOI:** 10.3389/fpsyt.2026.1708563

**Published:** 2026-03-13

**Authors:** Andrea Schlump, Bernd Feige, Swantje Matthies, Katharina von Zedtwitz, Isabelle Matteit, Kathrin Nickel, Katharina Domschke, Marco Reisert, Thomas Lange, Markus Heinrichs, Dominique Endres, Ludger Tebartz van Elst, Simon Maier

**Affiliations:** 1 Department of Psychiatry and Psychotherapy, Medical Center - University of Freiburg, Faculty of Medicine, University of Freiburg, Freiburg, Germany; 2 Department of Psychology, Laboratory for Biological Psychology, Clinical Psychology and Psychotherapy - University of Freiburg, Freiburg, Germany; 3 German Center for Mental Health (DZPG), Partner Site Berlin/Potsdam, Berlin, Germany; 4 Division of Medical Physics, Department of Diagnostic and Interventional Radiology, Medical Center - University of Freiburg, Faculty of Medicine, University of Freiburg, Berlin, Germany; 5 Department of Stereotactic and Functional Neurosurgery, Medical Center - University of Freiburg, Faculty of Medicine, University of Freiburg, Freiburg, Germany

**Keywords:** borderline personality disorder, electroencephalography, GABA, glutamate, IRDA/IRTA, NAA, spectroscopy

## Abstract

**Introduction:**

Previous studies have reported altered gamma-aminobutyric acid (GABA) and glutamate levels in borderline personality disorder (BPD), suggesting disruptions in excitatory-inhibitory neurotransmission. Electroencephalographic (EEG) research has indicated potential network hyperexcitability in BPD, evidenced by increased intermittent rhythmic delta and theta activity (IRDA/IRTA), which may reflect compensatory stabilization mechanisms. This study used multi-voxel magnetic resonance spectroscopic imaging (MRSI) to explore neurochemical abnormalities and their relationships with IRDA/IRTA, psychometric and neuropsychological measures.

**Methods:**

Sixty-six female patients diagnosed with BPD (mean age: 30.2 ± 9.7 years) and 29 age-matched female healthy controls (mean age: 27.8 ± 8.0 years) received spirally encoded 3D MRSI scans. GABA, glutamate plus glutamine (Glx), total creatine (tCr) and total N-acetylaspartate (tNAA) were quantified and reported as ratios relative to tNAA and/or tCr. The resulting spectroscopic images were analyzed using LCModel and FreeSurfer software, and IRDA/IRTA detection in a clinical EEG session was performed using independent component analysis. Metabolite ratios were analyzed using hierarchical linear mixed-effects models (ROIs nested within participants), with fixed effects of group or, in separate models, continuous predictors (IRDA/IRTA and psychometric/neuropsychological measures), ROI, and their interaction, and a subject-level random intercept. ROI-specific effects were quantified using estimated marginal means and within-ROI contrasts (emmeans).

**Results:**

No metabolite ratio showed a significant BPD vs. control difference. In BPD, IRDA/IRTA-related measures were positively associated with Glx/tCr and Glx/tNAA in the accumbens, and with Glx/tCr in the right caudal anterior cingulate cortex (cACC). BSL-supplement scores showed ROI-dependent associations with tCr/tNAA, with positive ROI-specific effects in the bilateral caudate, right pallidum, and right putamen. Alertness measures were linked to GABA/tCr, Glx/tNAA, and tCr/tNAA (including caudate, pallidum/putamen, cACC, and thalamus), while divided attention omissions and working-memory errors were associated with higher Glx/tCr in the cACC, isthmus cingulate, and hippocampus. ROI-dependent associations were also observed for IQ and verbal learning/recognition with GABA/tCr and tNAA/tCr.

**Discussion:**

While no robust group differences emerged, mixed-models using continuous predictors linked higher Glx ratios in nucleus accumbens/cACC to IRDA/IRTA and higher striatal tCr/tNAA to BPD symptom severity and neurocognitive performance. These exploratory multimodal signatures point to cortico-striato-limbic mechanisms in BPD and should be confirmed in larger samples.

## Introduction

Borderline personality disorder (BPD) is a debilitating psychiatric condition marked by emotional dysregulation, impulsivity, unstable self-identify, and difficulties in maintaining interpersonal relationships (1, 2). Affecting 1–3% of the general population, BPD constitutes a significant public health concern due to its associated psychosocial impact, high rates of comorbidity, self-injury and elevated suicide risk (3–7). Neuroimaging studies have identified abnormalities in fronto-limbic circuits critical for emotional processing and in temporoparietal circuits, including volume reductions in the amygdala and hippocampus (1, 8–11). Moreover, impaired glucose metabolism in these regions has been observed via [^18^F]-fluorodeoxyglucose positron emission tomography (FDG-PET) (12–15). Our previous findings demonstrated associations between alterations in cortical thickness and subcortical volume measures with symptom severity in BPD and slow-wave electroencephalography (EEG) activity, highlighting the putative involvement of the anterior cingulate cortex (ACC), temporal pole, and cerebellum in the neural underpinnings of emotional and cognitive dysfunction in individuals with BPD (16).

Within this framework, the balance between excitatory and inhibitory neurotransmission, mediated mainly by glutamate (Glu) and gamma-aminobutyric acid (GABA), is essential for cognitive and emotional stability (17–19). Altered Glu and GABA levels, as reported in MRS studies of the hippocampus (20), amygdalae (21, 22), prefrontal cortex (23), and ACC (24), suggest that disruptions in excitatory/inhibitory balance may underpin the neural network instability observed in BPD. Electrophysiological evidence further supports this notion: Increased intermittent rhythmic delta and theta activity (IRDA/IRTA) was observed in BPD and may represent a compensatory stabilization mechanism for hyperexcitable neural circuits (IRDA/IRTA) (25–27).

### The rationale of the study

Despite these advances, the relationship between fronto-limbic neurometabolite alterations and electrophysiological network hyperexcitability (IRDA/IRTA) in BPD remains unclear, as does their association with clinical symptomatology and cognitive dysfunction. To address these gaps, the present study utilizes a multi-voxel magnetic resonance spectroscopic imaging (MRSI) approach, which offers spatially resolved insights into neurometabolite distributions, to compare Glu, GABA, total creatine (tCr), total N-acetylaspartate (tNAA) in patients with BPD and healthy controls (HC). Based on previous studies (28, 29), we hypothesized that BPD patients will exhibit significant fronto-limbic neurometabolic alterations, including reduced tCr in the amygdala, decreased tNAA in the hippocampus, and increased glutamate levels in the ACC. These alterations were expected to be associated with elevated IRDA/IRTA rates as well as with BPD symptom severity and decreased cognitive performance.

## Methods

This study was part of a broader research project focusing on local network hyperexcitability. The quantitative brain structural findings of the BPD cohort and their correlations with IRDA/IRTA have been published previously (16). We obtained approval from the local ethics committee of the University Medical Center Freiburg, Germany (application no. EK-Freiburg: 209/18) according to the principles of the Declaration of Helsinki. The participants were enrolled after they had given their written informed consent.

### Patient assessment

**Figure 1:**
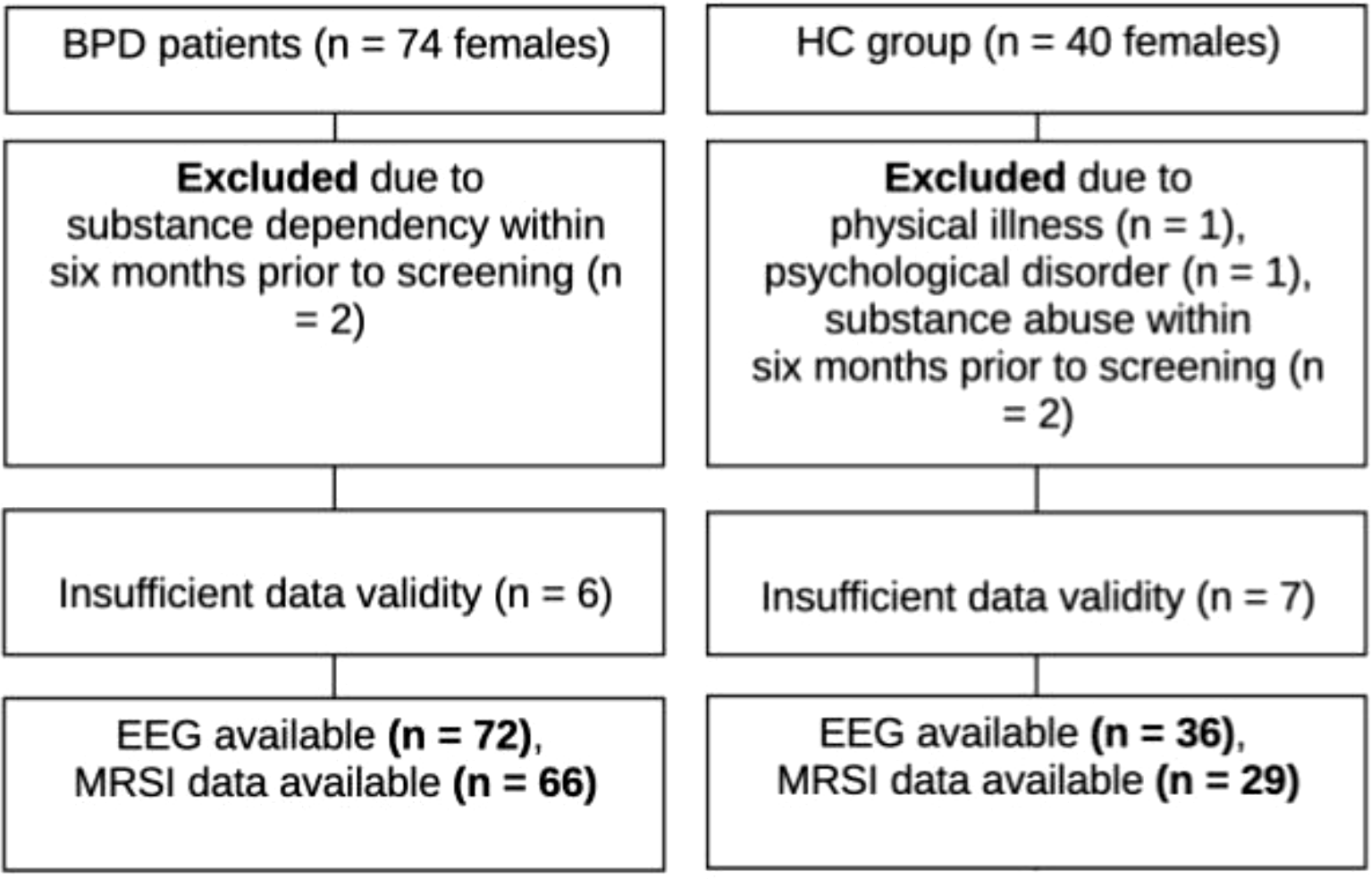
Participant recruitment diagram. BPD, borderline personality disorder; EEG, electroencephalography; HC, healthy controls; MRSI, magnetic resonance spectroscopic imaging; n, sample size.

The procedure has been described in detail elsewhere (16). In brief, female patients with BPD were enrolled from the BPD ward for dialectical behavior therapy (DBT) at the Department of Psychiatry and Psychotherapy, University Hospital Freiburg, Germany. This unit exclusively admits female patients aged >= 18 years. BPD was diagnosed by experienced clinicians according to the criteria of the International Classification of Diseases, 10th Revision (ICD-10). Participant recruitment adhered to the same procedure and utilized a subcohort (all with high-quality MRSI findings) of the sample of our recently published brain morphometric study (16). To confirm the diagnosis, all participants additionally fulfilled the DSM-IV criteria for BPD as assessed by the Structured Clinical Interview for DSM-IV Axis II Disorders (SCID-II; meeting at least 5 out of 9 criteria). Individuals with a lifetime diagnosis of bipolar disorder, schizophrenia, or acute psychotic symptoms were excluded. A history of substance dependence was also an exclusion criterion unless a maintained abstinence period for at least six months prior to screening could be confirmed. Further exclusion criteria included pregnancy, lactation, relevant physical illnesses that might interfere with assessments, lack of legal competence, and MRI contraindications (e.g., pacemakers) (**Figure 1**).

### Healthy control group assessment

The assessment of the adult (≥ 18 years) female healthy control group was also described in detail elsewhere (16).

### Sociodemographic, psychometric, and neuropsychological testing

Information on sociodemographics, comorbidities, and psychopharmacological medication was collected using a structured questionnaire. The psychometric and neuropsychological test batteries are described in a previous publication (16). Participants were not excluded due to missing questionnaire data or neuropsychological test results, if they met the general inclusion criteria. 

### Magnetic resonance imaging data acquisition

**Figure 2:**
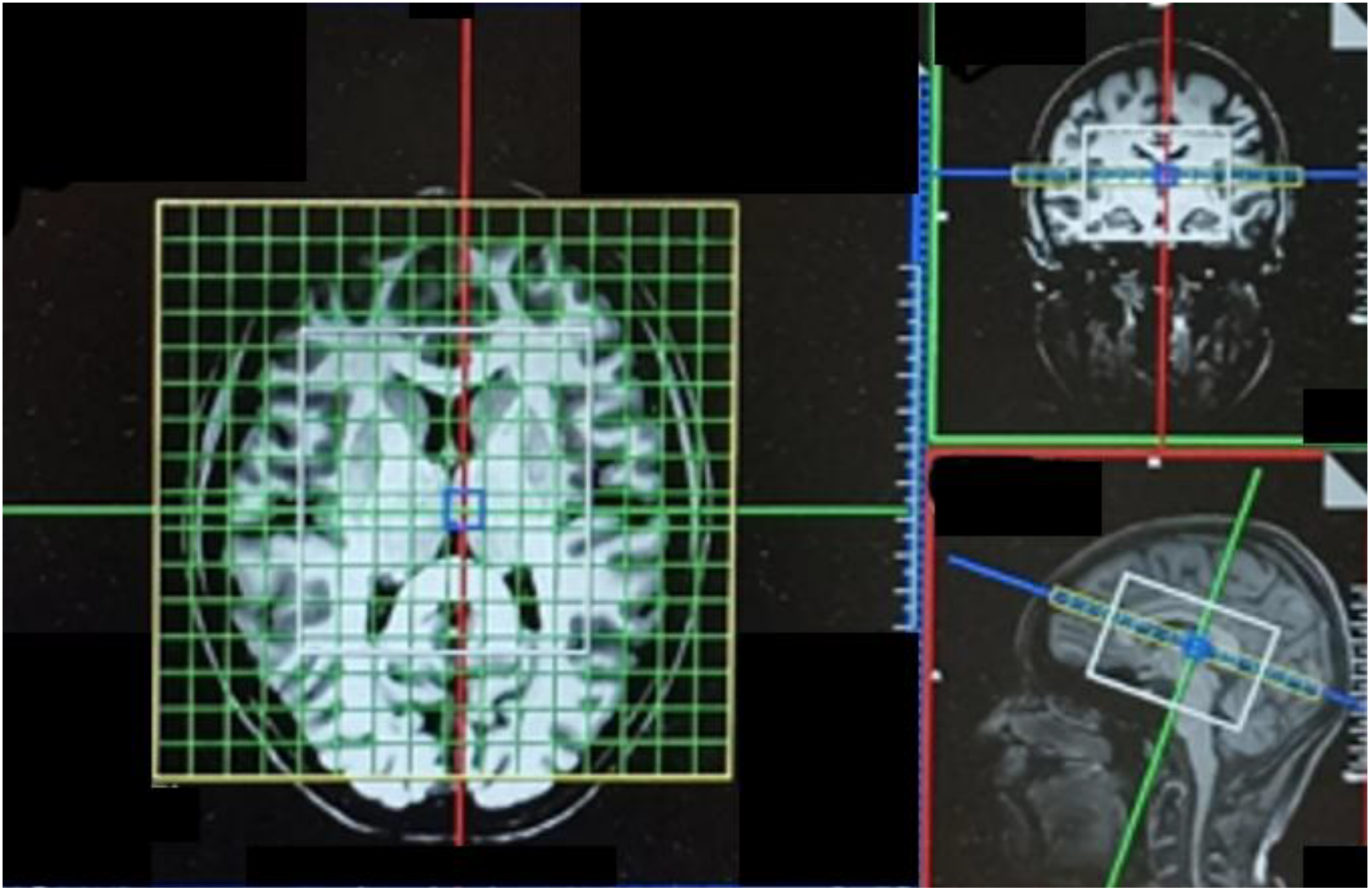
Setup of the 3D-MRSI measurement with the LASER voxel of interest (VOI, white box) covering the regions of interests and one exemplary transverse slice of the measured 3D-MRSI volume. The VOI was aligned along the anterior-to-posterior commissure line.

All MRI data were acquired during the same scanning session and time point as described in Schlump et al. (16), using a 3-Tesla MAGNETOM Prisma scanner (Siemens Healthineers^®^, Erlangen, Germany) with a 64-channel head coil at the Imaging Center, Department of Radiology, University Medical Center Freiburg. While our first publication (16) focused on the analysis of structural T1-weighted Magnetization-Prepared Rapid Gradient Echo (MPRAGE) data, the present study focused on magnetic resonance spectroscopic imaging (MRSI) data acquired within the same session. Structural images were obtained using the MPRAGE sequence with the following parameters: echo time (TE) = 4.11ms, field of view (FOV) = 256×256×160mm³, isotropic voxel size = 1×1×1mm³, and repetition time (TR) = 2000ms. These structural images enabled precise placement of the MRSI volume of interest (VOI) along the anterior-posterior commissure line, allowing for anatomically targeted MRS analysis. For multi-voxel MRS data acquisition, a spirally encoded three-dimensional MRSI sequence incorporating Mescher-Garwood (MEGA) and localized adiabatic selective refocusing (LASER) techniques (“MEGA-LASER 3D MRSI”) was adopted, as described by Bogner and colleagues (30). The acquisition parameters were set to a TR of 1600ms, TE of 68ms, flip angle of 90°, and 32 acquisition-weighted averages. To improve data quality, the sequence incorporated real-time adjustments for head motion and technical artifacts during the scans (30). The LASER method was specifically employed to mitigate B1 inhomogeneity, reduce chemical shift artifacts, and enhance the signal-to-noise ratio (SNR). Shimming and LASER localization were performed on a VOI measuring 80×90×80mm^3^, positioned within a FOV of 160×160×160mm^3^, which largely captured the whole brain (see **Figure 2**). Coverage of the cerebellum was not achieved in all measurements. A resolution of 10×10×10 voxels was used, yielding a nominal volume of 4.096cm³ per voxel. Following interpolation, the resolution was increased to 16×16×16 voxels, resulting in a refined voxel size of 1cm³. Gaussian MEGA editing pulses were alternately set at 1.9ppm and 7.5ppm for edit-on and edit-off scans with a bandwidth of 60 Hz and a pulse duration of 14.8ms. LASER localization pulses were applied with a center frequency of 3.0ppm. The protocol utilized a two-step phase cycling scheme, and 32 acquisition-weighted averages were recorded (30).

### Magnetic resonance spectroscopy analysis and quality checks

To process the MRSI data, a computational pipeline established by Bogner et al. (30) and Spurny et al. (31) has been adopted, which integrates a suite of software tools including MATLAB (version 2018a; MathWorks, Natick, MA, USA), Bash (Bourne-Again Shell), MINC (version 1.5; Medical Image NetCDF, McConnell Brain Imaging Center, Montreal, QC, Canada), the LCModel algorithm (version 6.3; Provencher, Oakville, ON, Canada) (31, 32), and FreeSurfer (version 6.0). This framework enables automated extraction and quantification of metabolite ratios from the spectra of individual regions of interest (ROIs). The data processing followed a two-step approach: First, LCModel was used voxel-wise to quantify metabolite concentrations from each spectrum, automatically estimating metabolite signals while also calculating the SNR, spectral line width, and Cramér-Rao lower bounds (CRLBs) as a measure of fitting accuracy (32). A voxel was considered valid if the CRLBs for tCr were below 30%. Second, metabolite concentrations estimated for each voxel were assigned to anatomical ROIs based on segmentations generated by FreeSurfer (for details see Spurny et al. (31)). The structural segmentation was conducted automatically using the *recon-all* command with default parameters on T1-weighted MPRAGE images to define ROI masks. These masks facilitated localized quantification of metabolite concentrations by averaging the valid voxel values within each ROI. ROIs with fewer than 80% valid voxels for tCr were excluded from the analysis. Similarly, regions with fewer than 80% valid voxels for other metabolites were omitted in the sub-analyses. Participants were excluded if above 50% of the ROIs had fewer than 80% valid voxels (33). Spectra suitable for subsequent analysis were obtained in 66 out of 72 eligible BPD patients (70 with sufficient MRI data) and 29 out of 36 HC (cf (16). see **Figure 1** and **Supplementary 1**-**3**). The volume of interest (VOI) was slightly increased compared to Spurny et al. (31) to mitigate effects of chemical shift displacement and achieve a more homogenous shim across all regions of interest. Unfortunately, the ACC did not yield spectra of sufficient quality for metabolite quantification. Consequently, this region was excluded from the final analysis, leaving a total of 20 ROIs. The remaining ROIs included the nucleus accumbens, cACC, caudate, corpus callosum, hippocampus, insula, isthmus of the cingulate gyrus, pallidum, putamen, and thalamus, analyzed bilaterally (left and right). Group-level analyses of metabolite ratios, including GABA/tNAA, GABA/tCr, Glx/tNAA, Glx/tCr, tNAA/tCr, and tCr/tNAA, were performed in R (see Statistical Analysis section) for the selected ROIs.

### EEG IRDA/IRTA analysis

Clinical EEG recordings were acquired during an 11-minute session using the standard 10/20 electrode placement system, which incorporated a 3-minute hyperventilation (HV) period. IRDA/IRTA events were identified using independent component analysis (ICA) implemented with a custom in-house software (available at https://github.com/berndf/avg_q (34)). The number of IRDA/IRTA events per minute prior to as well as during HV was quantified, and the IRDA/IRTA difference, i.e. the increase in IRDA/IRTA rates during compared to before HV, was calculated. The threshold for the IRDA/IRTA amplitude was set to > 1 µV as established in previous investigations (16, 33). This threshold was used to minimize floor effects: at this level, healthy participants show detections, with pathological states characterized primarily by an increased number of detections rather than their mere presence.

### Statistical analyses

For all statistical analyses, we used R software version 3.6.0 (R Foundation for Statistical Computing, Vienna, Austria). Group differences for all categorical variables (e.g., handedness and nicotine use) were assessed using the Fisher’s exact test. For continuous variables (e.g., age), the Welch two sample t-test was applied. To analyze the spectroscopy data, the normality of metabolite ratios was first assessed using the Shapiro-Wilk test in R (rstatix package) (35), supplemented by visual checks of histograms. These evaluations suggested that the metabolite ratios were not normally distributed.

To control for potentially confounding effects of the metabolite ratios – such as age, body mass index (BMI), smoking history (calculated as the number of cigarette packs per day multiplied by years of smoking), IQ from the CFT-20 R, and corresponding Cramér-Rao Lower Bounds (CRLBs), the Boruta feature selection algorithm (Boruta package) was applied. CRLBs were the sole covariate exhibiting a significant effect.

Minimum detectable effect sizes (MDEs) were computed for two-sided α = 0.05 and 80% power given the study sample sizes: (i) between-group comparisons (HC *n* = 29; BPD *n* = 66) and (ii) correlation analyses within the BPD group (*n* = 66) using the pwr package in R (36). Because our primary analyses use mixed-effects models with ROI nested within subjects, repeated ROI observations do not translate into an equivalent increase in independent sample size. Therefore, the subject-level MDEs provide a conservative benchmark.

### MR-spectroscopy group differences across ROIs

We screened for potentially influential covariates prior to the group analyses. For each metabolite ratio, data were first restricted to voxels meeting the ratio-specific quality criterion (valid voxels). We then fit a ratio-specific linear mixed-effects model using the mixed function of the afex package (37, 38) in R with group, ROI, and group × ROI as fixed effects and a random intercept for subject, and tested candidate covariates including centered BMI, pack-years, IQ, and the ratio-relevant CRLBs (numerator and denominator metabolites). CRLB terms showed consistent effects, whereas BMI, pack-years, and CFT-20 R were not significant in any ratio. Therefore, CRLBs were retained in the primary models.

ROI-level metabolite ratios were analyzed with linear mixed-effects models to account for the nesting of ROIs within participants. Models included fixed effects of group (BPD vs. HC), ROI, and their interaction (group × ROI) and a random intercept for subject (1|id). Continuous covariates were mean-centered prior to modelling. To control for spectral quality, metabolite-specific CRLB covariates (centered) were included (numerator and denominator CRLBs), together with age (centered). Models were fitted using afex with type III tests and Satterthwaite degrees-of-freedom approximation (38).

To obtain interpretable ROI-specific group differences, we computed estimated marginal means and pairwise group contrasts within each ROI using emmeans (39). Standardized effect sizes were expressed as Cohen’s d.

### Continuous predictor models

Associations between ROI-level metabolite ratios and continuous predictors (e.g., EEG, psychometry, neuropsychological measures) were tested using linear mixed-effects models with ROI-specific slopes. Models included fixed effects of ROI, the continuous predictor, and their interaction (ROI × predictor), plus a random intercept for subject (1|id). The predictor and all continuous covariates were mean-centered within the analysis dataset. As in the group models, ratio-specific CRLB covariates (centered; numerator and denominator) and age (centered) were included to control for spectral quality and demographic confounding. Models were fitted using afex:mixed (type III tests; Satterthwaite degrees-of-freedom approximation).

ROI-specific associations were calculated using estimated ROI-wise slopes of the predictor obtained via emmeans:emtrends. As a standardized effect size for each ROI-wise slope, we additionally report r, derived from the corresponding t-statistic and degrees of freedom.

### Multiple comparison correction

To control the false discovery rate, p-values were adjusted using the Benjamini–Hochberg (BH) procedure (40). We report two BH-adjusted p-values reflecting two inferential scopes:

- within-domain correction (*p_BHw_*), adjusting p-values across the set of predictors within a given predictor domain (e.g., the seven psychometric scales), and
- global correction (*p_BHg_*), adjusting p-values across all predictors multiplied by the number of metabolite ratios analyzed (e.g., predictors × six ratios).

Statistical significance was evaluated at p < 0.05 (two-sided) using these false discovery rate (FDR)-adjusted p-values.

Further, sensitivity analyses have been conducted by rerunning the mixed-effects models that were significant in the primary analyses to control for potential covariates (e.g., BMI, education, package-years, psychopharmacological medication, comorbidity).

To evaluate the potential impact of age and sex on the Test of Attentional Performance (TAP) data, a Boruta model was employed, with age being identified as a significant covariate. To control for the effects of age, a linear model (lm(TAP ~ age)) was applied to adjust the raw TAP scores to the mean age of participants. This allowed for comparability across the sample. All further analyses were based on these adjusted scores.

## Results

### Study cohort

Both the BPD and HC groups consisted exclusively of female participants and did not differ significantly in age (BPD: 30.2 ± 9.7, HC: 27.8 ± 8.0; *p* = 0.204). In contrast to the HC group, individuals with BPD exhibited significantly lower education levels (*p <* 0.001) and a higher rate of unemployment or retirement (*p <* 0.001; see **Table 1**). Among BPD patients, current or previous comorbidities were documented in 97% (n = 64; **Table 2**). None of the HC participants, but 55 BPD patients, were currently taking psychopharmacological medication (**Table 3**). Patients with BPD scored significantly higher on measures of BPD symptoms (BSL-23: 42.3 ± 19.9, *p <* 0.001; BSL-supplement: 3.29 ± 8.9, *p =* 0.025; IPDE Borderline: 3.26 ± 1.2, *p <* 0.001; IPO: 2.46 ± 0.7, *p <* 0.001), dissociative experiences (FDS-20: 16.4 ± 14.5, *p <* 0.001), and difficulties in emotion regulation (Difficulties in Emotion Regulation Scale, DERS: 54.3 ± 24.7, *p <* 0.001). All psychometric and neuropsychological differences are summarized in **Table 4**.

**Table 1:** Sociodemographic characteristics of the borderline personality disorder and healthy control group.

Sociodemographic characteristic	BPD, N = 66^1^	HC, N = 29^1^	p-value^2^
Age	30.21 ± 9.7 (N = 66)	27.76 ± 8.0 (N = 29)	0.204
Education level			<0.001
University degree	9 (14%; N = 64)	15 (54%; N = 28)	
High degree	24 (38%; N = 64)	11 (39%; N = 28)	
Middle degree	27 (42%; N = 64)	2 (7.1%; N = 28)	
Low degree	3 (4.7%; N = 64)	0 (0%; N = 28)	
Other qualification	1 (1.6%; N = 64)	0 (0%; N = 28)	
Employment status			<0.001
Unemployed	23 (36% N = 64)	0 (0%; N = 28)	
Retired	6 (9.4% N = 64)	0 (0%; N = 28)	
Apprentice	2 (3.1% N = 64)	2 (7.1%; N = 28)	
Student	10 (16% N = 64)	15 (54%; N = 28)	
Part-time job	19 (30% N = 64)	6 (21%; N = 28)	
Full-time job	4 (6.3%; N = 64)	5 (18%; N = 28)	
Marital status			0.683
Single/unwed	54 (84%; N = 64)	26 (93%; N = 28)	
Married	5 (7.8%; N = 64)	1 (3.6%; N = 28)	
Divorced	5 (7.8%; N = 64)	1 (3.6%; N = 28)	
Handedness			0.275
Both	3 (4.7%; N = 64)	0 (0%; N = 28)	
Left	4 (6.3%; N = 64)	0 (0%; N = 28)	
Right	57 (89%; N = 64)	28 (100%; N = 28)	
BMI	26.7 ± 7.1 (N = 62)	21.4 ± 2.0 (N = 28)	<0.001
Tobacco	25 (38%; N = 65)	5 (17%; N = 29)	0.055
Package years	2.69 ± 6.2 (N = 62)	0.25 ± 0.8 (N = 29)	0.003

^1^Mean ± SD; n (%; N = N Non-missing. ^2^Welch Two Sample t-test; Fisher’s exact test. BMI, body mass-index; BPD, borderline personality disorder; HC, healthy controls; N, sample size; p-value, probability value; SD, standard deviation.

**Table 2:** Comorbid conditions in the borderline personality disorder and healthy control group.

Comorbid disorder	BPD, N = 66^1^	HC, N = 29^1^
Current or recent depression	54 (81.8%)	0 (0%)
Anxiety disorder	16 (24.2%)	0 (0%)
PTSD	16 (24.2%)	0 (0%)
Autism	3 (4.5%)	0 (0%)
ADHD	31 (47%)	0 (0%)

ADHD, attention-deficit/hyperactivity disorder; BPD, borderline personality disorder; HC, healthy controls; N, sample size; PTSD, post-traumatic stress disorder. ^1^n (%).

**Table 3:** Psychiatric medication of the borderline personality disorder and healthy control group.

Status	Psychopharmacological medication	BPD, N = 66^1^	HC, N = 29^1^
Current	Antidepressants	44 (66.6%)	0 (0%)
	Typical antipsychotics	5 (7.6%)	0 (0%)
	Atypical antipsychotics	26 (39.4%)	0 (0%)
	Mood stabilizers*	7 (10.6%)	0 (0%)
	Benzodiazepines	0 (0%)	0 (0%)
	ADHD medication	12 (18.2%)	0 (0%)
Former	Antidepressants	44 (66.7%)	0 (0%)
	Typical antipsychotics	7 (10.6%)	0 (0%)
	Atypical antipsychotics	24 (36.4%)	0 (0%)
	Mood stabilizer*	6 (9.1%)	0 (0%)
	Benzodiazepines	14 (21.2%)	0 (0%)
	ADHD medication	12 (18.2%)	0 (0%)

*Lamotrigine was prescribed to one patient (without a history of epilepsy or seizures) for the treatment of an affective disorder. Pregabalin was administered to two patients—also without epilepsy or seizures—for indications including polyneuropathy and generalized anxiety disorder. Values are presented as n (%). ADHD, attention-deficit/hyperactivity disorder; BPD, borderline personality disorder; HC, healthy controls; N, sample size.

**Table 4:** Psychometric and neuropsychological assessments in individuals with borderline personality disorder and healthy controls.

Psychometry	p-value^1^	Neuropsychological test	p-value^1^
BSL-23	<0.001	CFT-20 R	0.013
BSL-Supplement	0.025	MWTB	0.566
IPDE Borderline	<0.001	VLMT Learning	0.065
IPO	<0.001	VLMT False positive	0.672
DERS	<0.001	VLMT Perservations	0.848
FDS-20	<0.001	VLMT Recognition	0.359
WURS-k	<0.001	VLMT Consolidation	0.263
ADHD-Checklist	<0.001	Alertness no warning tone	0.465
BDI-II	<0.001	Alertness warning tone	0.786
EQ	0.001	Phasic alertness	0.310
AQ	<0.001	Working memory mistakes	0.220
SCL-90-R Hostility	<0.001	Working memory missings	0.057
SCL-90-R Anxiety	<0.001	Mental flexibility	0.116
SCL-90-R Depression	<0.001	Divided attention mistakes	0.115
SCL-90-R Somatization	<0.001	Divided attention missings	0.043
SCL-90-R Obsessive-compulsive	<0.001		
SCL-90-R Interpersonal sensitivity	<0.001		
SCL-90-R Phobic anxiety	<0.001		
SCL-90-R Paranoid ideation	<0.001		
SCL-90-R Psychoticism	<0.001		
STAI-G Trait	<0.001		

^1^Welch Two Sample t-test; Fisher’s exact test. ADHD-CL, attention-deficit/hyperactivity disorder-checklist for DSM-IV; AQ, Autism Spectrum Quotient; BDI-II, Beck Depression Inventory II; BSL, Borderline-Symptom List; BPD, borderline personality disorder; CFT-20 R, Culture Fair Intelligence Testing; DERS, Difficulties in Emotion Regulation Scale; EQ, Cambridge Behavior Scale-40; FDS-20, Dissociation Experience Scale; HC, healthy controls; MWT-B, Multiple-choice Word Test; N, sample size; p-value, probability value; SCL-90-R, Symptom-Checklist; SD, standard deviation; STAI-G, State-Trait Anxiety Inventory; VLMT, Verbal Learning and Memory Test; WURS, Wender Utah Rating Scale.

Given the available sample sizes (HC *n* = 29; BPD *n* = 66), the minimum detectable effect at α = 0.05 and 80% power was d = 0.63 for between-group comparisons and |r| = 0.34 for correlations computed within the BPD group (*n* = 66).

### MR-spectroscopy group differences across ROIs

**Figure 3:**
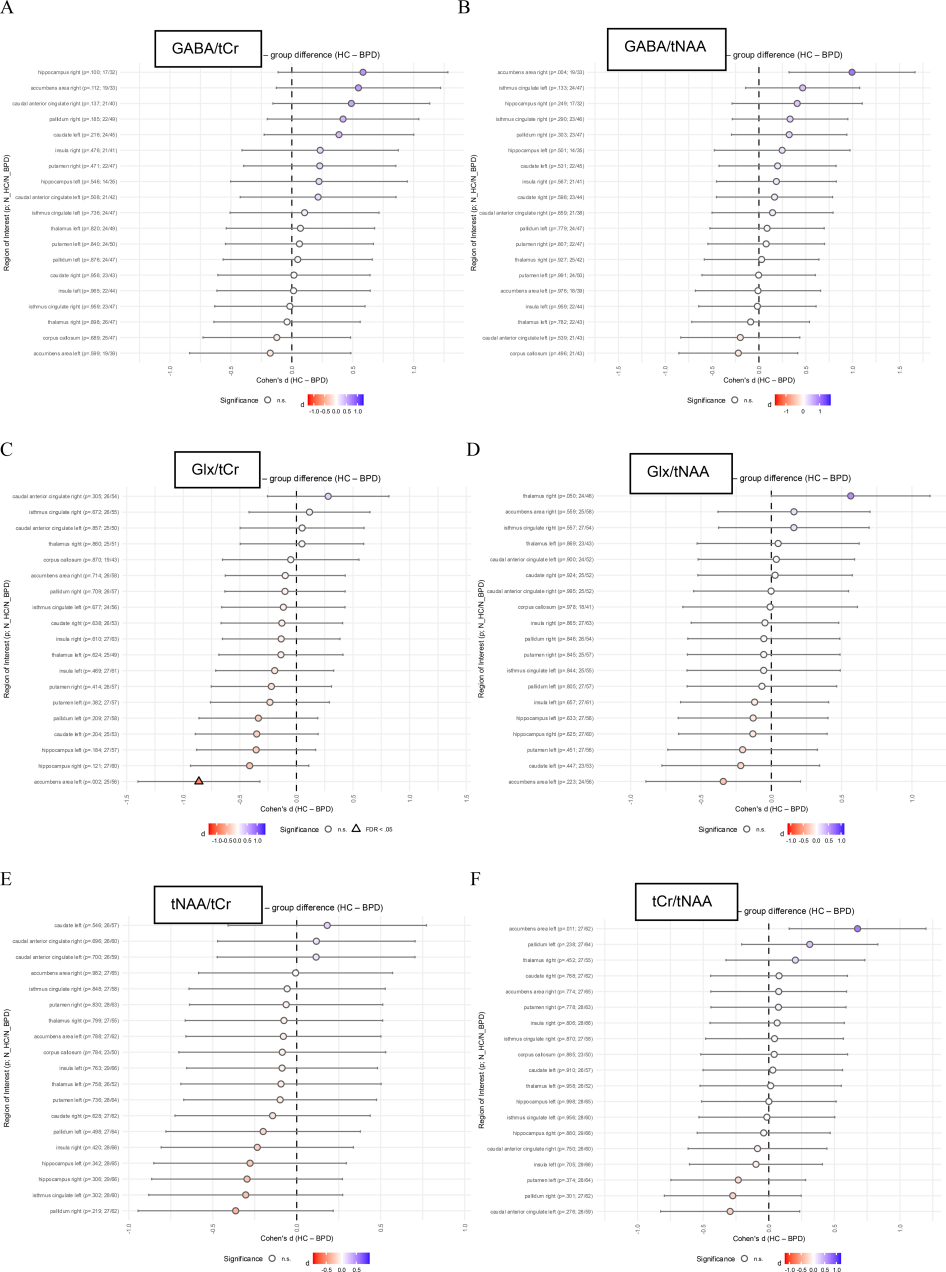
ROI-specific group differences (HC-BPD) in metabolite ratios: **(A)** GABA/tCr, **(B)** GABA/tNAA, **(C)** Glx/tCr, **(D)** Glx/tNAA, **(E)** tNAA/tCr, **(F)** tCr/tNAA. The linear mixed-effects group models provided no evidence for a group effect or a group × ROI interaction for all GABA, Glx, tCr, and tNAA ratios. Exploratory *post-hoc* within-ROI contrasts indicated higher Glx/tCr ratios in the in left accumbens. Points show Cohen’s *d* (HC-BPD) with 95% CIs from mixed-effects models; the dashed line indicates no group difference. Triangles indicate ROIs surviving Benjamini–Hochberg FDR correction (*p*_BH_ < 0.05); circles indicate non-significant ROIs. ROI labels include the unadjusted *p*-value and sample sizes (N_HC/N_BPD). BPD, borderline personality disorder; GABA, Gamma-aminobutyric acid; Glx, glutamate and glutamine; HC, healthy controls; tCr, total creatine; tNAA, total N-acetylaspartate.

Neurometabolite ratios were compared between BPD patients and HC in different brain regions. The linear mixed-effects group models provided no evidence for a group effect or a group × ROI interaction for all GABA, Glx, tCr, and tNAA ratios. Exploratory *post-hoc* within-ROI contrast analyses are summarized for descriptive purposes in **Figure 3**.

### Continuous predictor models

#### Association of metabolite ratios with EEG IRDA/IRTA in BPD patients

**Figure 4:**
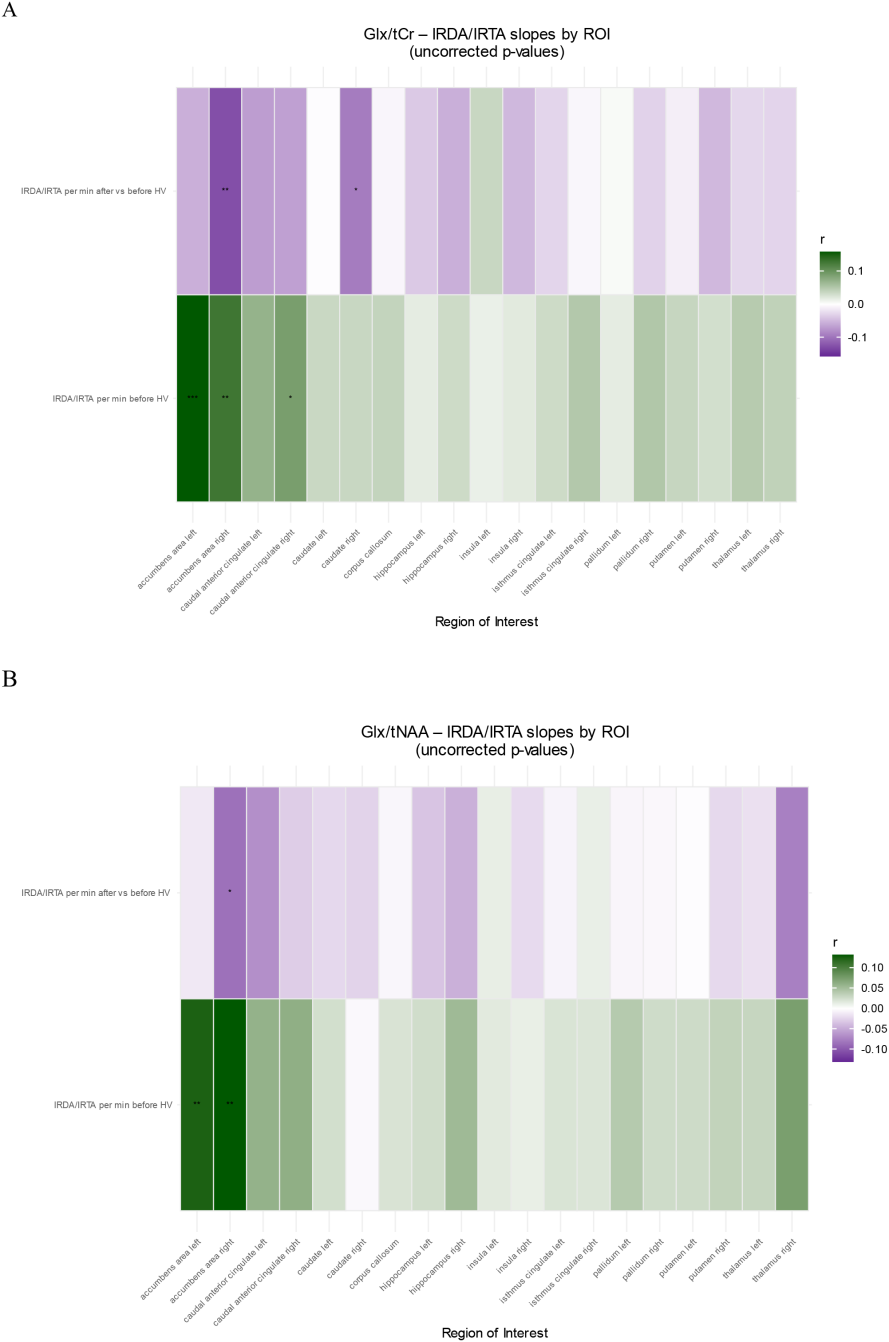
ROI-wise associations between IRDA/IRTA and metabolite ratios in patients with borderline personality disorder: **(A)** Glx/tCr; **(B)** Glx/tNAA shown as heatmaps. Columns represent ROIs and rows represent EEG predictors (IRDA/IRTA per min before hyperventilation; after vs. before hyperventilation). Tile color encodes the standardized effect size *r* from ROI × predictor mixed-effects models (green = positive, purple = negative; intensity = |r|). Asterisks indicate BH–FDR–adjusted significance (*p__BH_ < 0.05; **p__BH_ < 0.01; ***p_BH < 0.001). HV, hyperventilation; IRDA/IRTA, intermittent rhythmic delta/theta activity; Glx, glutamate+glutamine; tCr, total creatine; tNAA, total N-acetylaspartate.

In ROI-by-predictor mixed-effects models, higher IRDA/IRTA per minute before HV was associated with higher Glx/tCr (p = 0.016) and Glx/tNAA (p = 0.036), independent of ROI. In follow-up ROI-wise analyses, IRDA/IRTA was positively associated with Glx/tCr in the left (p < 0.001) and right accumbens (p = 0.002), and right cACC (p = 0.022), and with Glx/tNAA in the left (p = 0.002) and right accumbens (p < 0.001). The IRDA/IRTA difference score showed no significant associations with metabolite ratios and no IRDA/IRTA difference × ROI interaction. In **Figure 4** all post-hoc findings are reported, including alterations in IRDA/IRTA differences in which the primary model was not significant.

#### Association of metabolite ratios with psychometry in BPD patients

**Figure 5:**
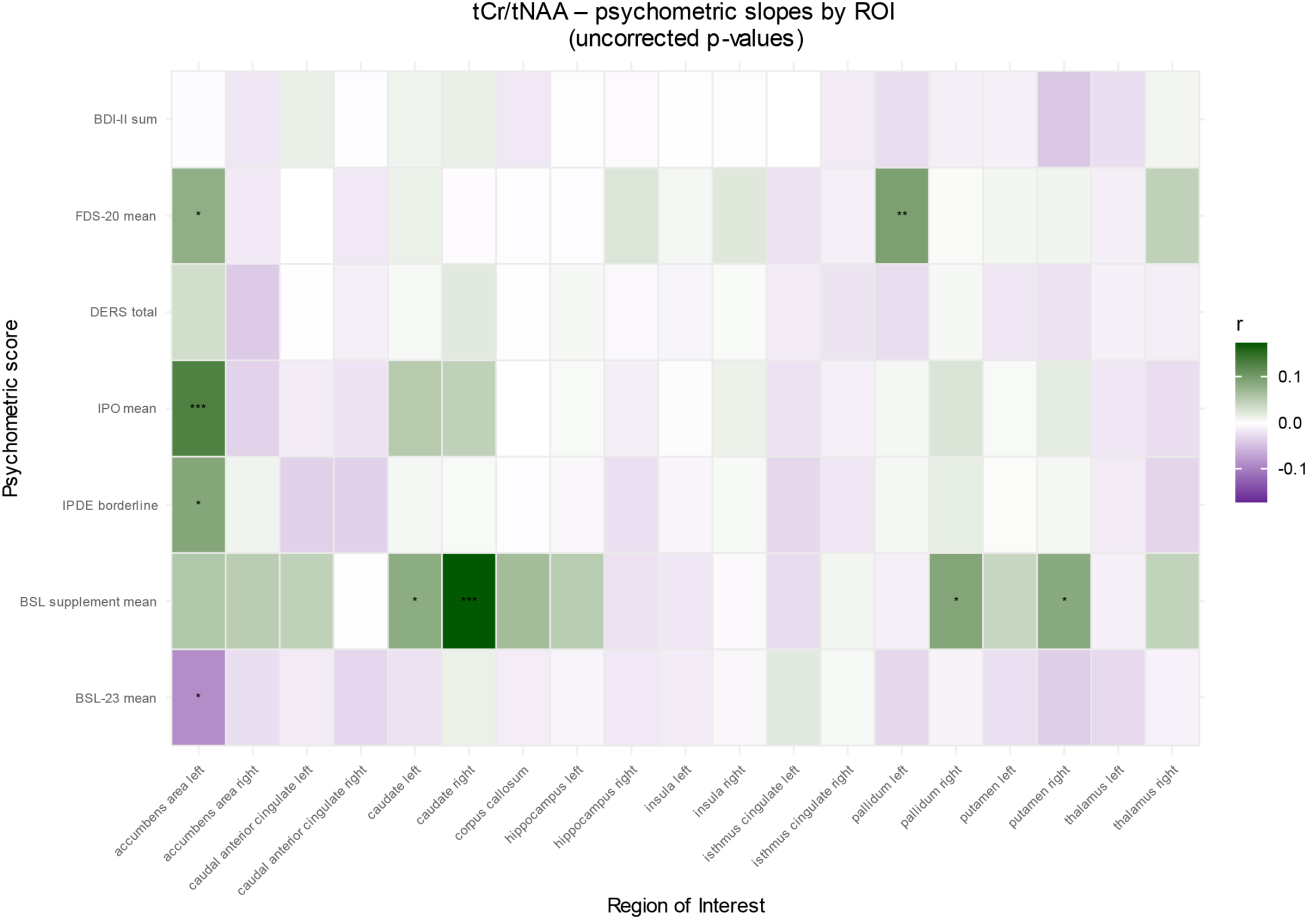
ROI-wise associations between psychometry and metabolite ratios in patients with borderline personality disorder for tCr/tNAA. Columns represent ROIs and rows represent neuropsychological test predictors. Tile color encodes the standardized effect size *r* from ROI × predictor mixed-effects models (green = positive, purple = negative; intensity = |r|). Asterisks indicate BH–FDR–adjusted significance (*p__BH_ < 0.05; **p__BH_ < 0.01; ***p_BH < 0.001). BDI, Beck’s depression inventory; BSL, borderline symptom list; DERS, difficulties in emotion regulation scale; FDS, Freiburger Dissoziationsskala; IPDE, International Personality Disorder Examination; IPO, Inventory of Personality Organization.

In ROI-by-predictor mixed-effects models, mean BSL-supplement scores were associated with tCr/tNAA (p = 0.040), with a significant ROI × BSL-supplement interaction (p = 0.001). In follow-up ROI-wise robust analyses, BSL-supplement was positively associated with tCr/tNAA in the left caudate (p = 0.018), right caudate (p < 0.001), right pallidum (p = 0.011), and right putamen (p = 0.016). No other psychometric scores showed significant associations or ROI interactions. In **Figure 5** all *post-hoc* findings are reported, including alterations in all psychometric tests in which the primary model was not significant.

#### Association of metabolite ratios with neuropsychological testing in BPD patients

For GABA/tCr, alertness (warning tone) showed a significant main effect (p = 0.046), and significant predictor × ROI interactions were observed for IQ (p = 0.003), VLMT learning (p = 0.001), and VLMT recognition (p = 0.027). In follow-up ROI-wise analyses, reduced alertness (warning tone) was associated with higher GABA/tCr in the left caudate (p = 0.025), left pallidum (p = 0.009), and left putamen (p = 0.003). Higher GABA/tCr was also associated with better IQ in the left caudate (p < 0.001), poorer VLMT learning in the left caudate (p < 0.001), and better VLMT recognition in the left caudate (p = 0.003).

For Glx/tCr, significant predictor × ROI interactions were found for divided-attention omissions (p = 0.046) and working-memory errors (p = 0.009). In follow-up ROI-wise analyses, more divided-attention omissions were associated with higher Glx/tCr in the left cACC (p = 0.012), right hippocampus (p = 0.019), and right isthmus cingulate (p = 0.045), and more working-memory errors were associated with higher Glx/tCr in the left cACC (p < 0.001), right cACC (p = 0.004), and right isthmus cingulate (p = 0.039).

For Glx/tNAA, significant alertness × ROI interactions were observed for both conditions (no warning tone: p < 0.001; warning tone: p < 0.001), and alertness main effects were also significant (no warning tone: p = 0.028; warning tone: p = 0.020). In follow-up ROI-wise analyses, higher alertness (no warning tone) was associated with higher Glx/tNAA in the left cACC (p = 0.015) and right thalamus (p < 0.001), and higher alertness (warning tone) was associated with higher Glx/tNAA in the left cACC (p = 0.027) and right thalamus (p < 0.001).

For tCr/tNAA, alertness (no warning tone) showed both a main effect (p = 0.035) and an ROI-dependent effect (p = 0.012); ROI-dependent effects were also observed for alertness with warning tone (p = 0.023) and phasic alertness (p = 0.006). In follow-up ROI-wise analyses, better alertness (no warning tone) was associated with higher tCr/tNAA in the left caudate (p = 0.043), right caudate (p = 0.003), left pallidum (p = 0.040), right pallidum (p = 0.023), right putamen (p = 0.013), and right thalamus (p < 0.001). Better alertness (warning tone) was associated with higher tCr/tNAA in the left pallidum (p = 0.020), right putamen (p = 0.019), and right thalamus (p < 0.001). Higher tCr/tNAA was also associated with better phasic alertness in the left accumbens area (p < 0.001), left caudate (p = 0.017), and right caudate (p = 0.006).

**Figure 6:**
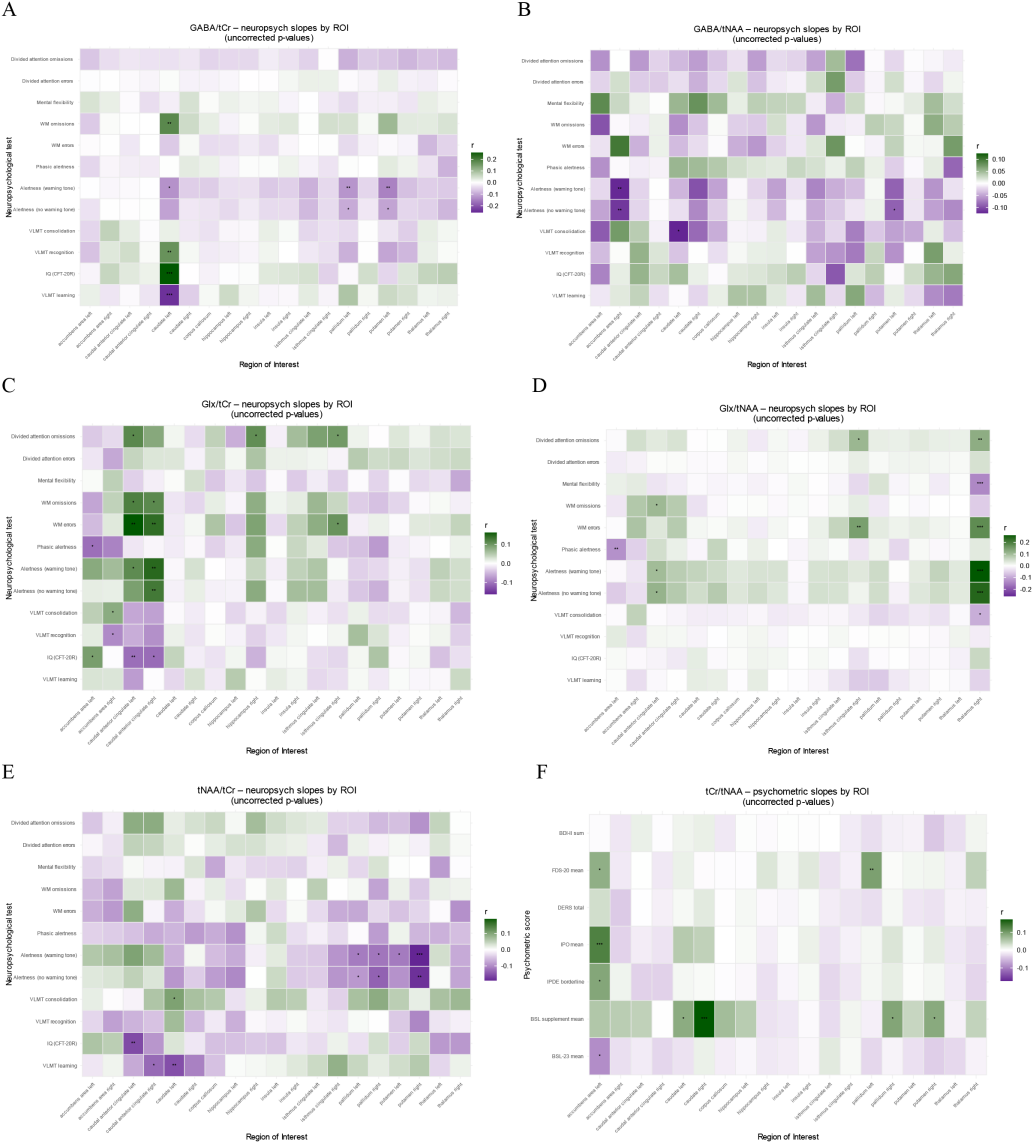
ROI-wise associations between neuropsychological tests and metabolite ratios in patients with borderline personality disorder: **(A)** GABA/tCr, **(B)** GABA/tNAA, **(C)** Glx/tCr, **(D)** Glx/tNAA, **(E)** tNAA/tCr, **(F)** tCr/tNAA. Columns represent ROIs and rows represent neuropsychological test predictors. Tile color encodes the standardized effect size *r* from ROI × predictor mixed-effects models (green = positive, purple = negative; intensity = |r|). Asterisks indicate BH–FDR–adjusted significance (*p__BH_ < 0.05; **p__BH_ < 0.01; ***p_BH < 0.001). CFT-20 R, Culture Fair Intelligence Testing; IQ, intelligence quotient; VLMT, Verbal Learning and Memory Test; WM, working memory.

For tNAA/tCr, significant predictor × ROI interactions were observed for alertness (no warning tone: p = 0.001; warning tone: p < 0.001), VLMT learning (p = 0.001), and IQ (p = 0.037). In follow-up ROI-wise analyses, poorer alertness (no warning tone) was associated with higher tNAA/tCr in the left pallidum (p = 0.038), right pallidum (p = 0.017), and right putamen (p = 0.002), and poorer alertness (warning tone) was associated with higher tNAA/tCr in the left pallidum (p = 0.023), right pallidum (p = 0.034), left putamen (p = 0.042), and right putamen (p < 0.001). Higher tNAA/tCr was also associated with poorer IQ in the left cACC (p = 0.003) and poorer VLMT learning in the left caudate (p = 0.005) and right cACC (p = 0.020). In **Figure 6** all post-hoc findings are reported, including alterations in neuropsychological tests in which the primary model was not significant. A more detailed description of the continuous predictor models can be found in the **Supplementary 4**.

### Sensitivity analyses for BPD

To evaluate whether the reported significant findings were robust to potential confounding, we conducted sensitivity analyses for BPD by rerunning the mixed-effects models that were significant in the primary analyses, each time adding a different potential covariate (e.g., BMI, education, package-years, psychopharmacological medication, comorbidities) to the model. Overall, the pattern of results was largely consistent across sensitivity specifications. However, several effects exhibited covariate dependence. In the EEG models, the association between IRDA/IRTA per minute before HV and Glx/tNAA (*p =* 0.036) was attenuated after adjustment for current typical neuroleptic use and no longer reached statistical significance (*p =* 0.065). Likewise, in the psychometry models, the main effect of the BSL-supplement mean score on tCr/tNAA became non-significant when controlling for current typical neuroleptic use (*p =* 0.221) and current atypical neuroleptic use (*p =* 0.054). In neuropsychological models, multiple associations were affected: the main effect of alertness (warning tone) on GABA/tCr became non-significant after controlling for package-years (*p =* 0.052), depression (*p =* 0.062) and ADHD (*p =* 0.062). For Glx/tNAA, the main effects for alertness did not remain significant when accounting for typical neuroleptic medication (no warning tone, *p =* 0.237; warning tone, *p =* 0.089). The ROI-wise effect for tCr/tNAA and alertness (no warning tone) became non-significant after adjusting for BMI (*p =* 0.054). Similarly, the main effect for tCr/tNAA and alertness (no warning tone) did not reach significance after controlling for typical neuroleptic use (*p =* 0.083). Also, the tCr/tNAA interaction between ROI and phasic alertness, along with its main effect became non-significant after adjusting for package years (*p =* 0.147 and *p =* 0.113, respectively). The main effect also did not reach significance following adjustment for typical neuroleptic use (*p =* 0.111). For tNAA/tCr, the interaction effects between ROI and IQ (CFT-20 R) and ROI with VLMT learning became non-significant after BMI adjustment (*p =* 0.067 and *p =* 0.075, respectively). The interaction effect for ROI and VLMT learning did not show significance after adjusting for education (*p =* 0.078) and package years (*p =* 0.09). Additionally, the interaction effect between ROI and IQ attenuated after controlling for package years (*p =* 0.065).

## Discussion

This multimodal study is the first to integrate multi-voxel MRSI, EEG, psychometric and neuropsychological assessments in females with BPD.

### Group comparisons

Mixed-effects models showed no main effect of group and no group-by-ROI interaction across metabolite ratios, indicating that robust, regionally consistent neurometabolite abnormalities were not detectable in this cohort of females with BPD using multivoxel MRSI. Accordingly, the data did not support our *a priori* predictions, which were derived from prior single-voxel MRS reports of fronto-limbic alterations (reduced amygdala tCr, decreased hippocampal tNAA, and elevated ACC glutamate) (22, 28, 29). Notably, the rostral ACC (rACC) could not be evaluated because spectral quality criteria were not met, precluding a direct test of the hypothesized ACC glutamatergic alteration and limiting coverage of a key region implicated in BPD pathophysiology. Finally, with a minimum detectable effect of approximately d = 0.63, the study was primarily sensitive to moderate-to-large between-group differences; thus, smaller effects or effects restricted to clinically defined subgroups—may not have been detected.

### Association of metabolite ratios with EEG IRDA/IRTA in BPD patients

The nucleus accumbens serves a key striatal hub within cortico-striato-limbic circuits, integrating prefrontal regulatory regions (ACC, dorsolateral PFC) with limbic structures (amygdala, hippocampus) to mediate reward processing, reinforcement learning, motivation, and impulse control (41, 42). In BPD, the nucleus accumbens has been implicated in emotional dysregulation and impulsivity, core symptoms of the disorder (43, 44). Its functioning is critically modulated by glutamatergic signaling, which influences the balance of excitation and inhibition across these networks (45). In this study, higher glutamate ratios in the nucleus accumbens and cACC were linked to increased rates of IRDA/IRTA measured before HV, indicating network hyperexcitability. While the rACC primarily contributes to affective processing and emotional evaluation, the cACC has been linked to conflict and error monitoring as well as inhibitory control, thereby modulating striatal impulsivity (28, 46, 47). These associations suggest that IRDA/IRTA may, at least in part, reflect glutamatergic dysregulation within the nucleus accumbens and cACC, supporting the hypothesis of a local excitation-inhibition imbalance (25, 48). In summary, disrupted glutamatergic signaling in the nucleus accumbens, a pivotal area of the brain’s reward system, extending to right cACC, may drive electrophysiological network instabilities that might contribute to the impulsivity and emotional instability observed in BPD (49). These findings underscore the importance of further research into the neurochemical and electrophysiological mechanisms underlying BPD.

### Association of metabolite ratios with psychometry in BPD patients

Primary mixed-effects models revealed positive associations between tCr/tNAA ratios and mean BSL-supplement score, with significant effects localized in the left and right caudate, right pallidum, and right putamen, which constitute striatal regions (50, 51). The BSL-supplement is used to assess dysfunctional behaviors like self-harm and impulsive outburst (52–54). Previous MRS research on BPD has primarily focused on limbic structures, particularly the amygdala, where reduced tNAA and tCr concentrations have been reported (22). The present findings extend this evidence by demonstrating that elevated tCr/tNAA ratios in the striatum may be associated with clinical symptoms of BPD. Higher tCr/tNAA ratios could reflect either elevated creatine levels, which is a marker of cerebral energy metabolism, or reduced NAA levels, a marker of neuroaxonal damage or mitochondrial dysfunction (55–59). Especially, elevated tCr/tNAA ratios might reflect bioenergetic demands of sustained striatal engagement in the context of endured emotional dysregulation in BPD (1, 19, 60, 61). However, these findings should be interpreted as cautious because of the explorative approach.

### Association of metabolite ratios with neuropsychological testing in BPD patients

The present study investigated region-specific associations between brain metabolite ratios and cognitive performance including alertness, attention, working memory, fluid intelligence, verbal learning and memory. Several significant associations were identified between neurometabolite ratios and different neuropsychological test results. Our exploratory approach suggests that MRSI may be effective for capturing different neuropsychological domains, particularly in contexts requiring region- and metabolite-specific resolution of GABAergic (striatal: impaired alertness/improved IQ) and glutamatergic (cingulate: attention/working memory deficits) effects on cognitive subdomains. The involvement of these neurometabolites, and their relative balance, in modulating cognitive processes is supported by previous studies (62–65). This could be a major strength of the multivoxel MRSI approach, which allows to analyze multiple regions in parallel rather than solely single voxels as in most previous studies (22, 29).

### Methodological considerations and limitations

This study has several limitations. Data loss occurred due to technical issues, and insufficient spectral validity resulted in a smaller sample of BPD patients with MRSI data compared to our previously published EEG (N = 72) and structural MRI cohort (N = 70; cf (16)). Additionally, not all regions could be reliably measured, resulting in smaller samples for specific regions (**Supplementary 3**). Absolute metabolite quantification was unavailable due to the absence of unsuppressed water reference scans, which would have extended acquisition time beyond clinical feasibility. While metabolite ratios effectively reduce between-subject variability from coil loading and scaling factors, they introduce interpretational ambiguity as ratio changes may reflect alterations in numerator, denominator, or both (66). Future studies should prioritize water-referenced absolute quantification. Compared with single-voxel MRS, multivoxel MRSI trades spatial coverage for per-voxel SNR; smaller effective voxel volumes and additional correction for multiple ROIs may reduce sensitivity to subtle effects. Finally, MRS predominantly reflects bulk tissue metabolite pools and cannot isolate synaptic/extracellular dynamics. Given the moderate sample size (n = 95) and the simultaneous analysis of multiple ROIs and metabolite ratios, this exploratory design carries a potential risk of overfitting, underscoring the need for replication in larger cohorts. The female BPD cohort (n = 66; predominantly receiving specialized inpatient DBT treatment) versus smaller unmedicated controls (n = 29) enhances internal validity for severe cases. However, the study design compromises external validity by limiting generalizability to male patients, less severe BPD presentation, and pharmacologically matched cohorts (1, 67, 68). Sample size asymmetry and medication differences may have reduced statistical power. Especially neuroleptic medication (e.g., with low-potency, typical neuroleptics which can be used against insomnia or to reduce inner tension) could have influenced our results. Several associations proved sensitive to covariates (neuroleptics, BMI, education, package-years, comorbidities), necessitating cautious interpretation. Most notably, the rACC could not be measured with sufficient data quality, precluding evaluation of our hypothesis regarding glutamate changes in this region.

## Conclusions

In summary, while no significant group differences in neurometabolite ratios were observed between BPD patients and HC, our multimodal approach emphasizes the potential of integrating MRSI with EEG to investigate the neurobiology of BPD. Future research employing multimodal approaches is essential to deepen our understanding of the neurobiological underpinnings of BPD.

## Data Availability

The raw data supporting the conclusions of this article will be made available by the authors, without undue reservation.
