# Supplementary material for: Multi-voxel MR-spectroscopy signatures and associations with EEG network hyperexcitability and clinical symptomatology in borderline personality disorder
Source: Front Psychiatry. 2026 Mar 13;17:1708563. doi: 10.3389/fpsyt.2026.1708563 (PMC13022906; doi:10.3389/fpsyt.2026.1708563)
Supplement: Supplementary file 1 [file DataSheet1.pdf]

1. Supplement: Mean CRLB per ROI and metabolite (valid ROIs/voxels): mean ± SD

ROI	GABA	Glx	tNAA	tCr
accumbens area left	12.4 ± 5.0	10.0 ± 4.9	7.5 ± 4.1	8.5 ± 4.1
accumbens area right	12.2 ± 4.5	10.5 ± 5.2	8.4 ± 4.7	8.4 ± 3.9
caudal anterior cingulate left	11.0 ± 3.1	9.4 ± 3.4	5.3 ± 3.0	5.9 ± 3.1
caudal anterior cingulate right	11.3 ± 3.2	9.1 ± 3.0	5.5 ± 3.5	6.0 ± 3.7
caudate left	10.3 ± 3.2	8.5 ± 3.0	5.9 ± 2.9	6.4 ± 2.8
caudate right	10.5 ± 3.0	8.7 ± 2.8	7.0 ± 3.2	7.3 ± 3.2
corpus callosum	9.4 ± 2.7	7.5 ± 2.9	4.4 ± 2.9	4.8 ± 2.4
hippocampus left	11.6 ± 2.5	8.8 ± 3.7	4.7 ± 2.7	5.8 ± 3.0
hippocampus right	11.8 ± 3.2	9.0 ± 3.5	4.8 ± 3.1	5.9 ± 2.9
insula left	12.1 ± 2.4	8.6 ± 2.7	5.1 ± 2.5	5.8 ± 2.4
insula right	11.9 ±	8.2 ± 2.3	5.1 ± 2.6	5.6 ± 2.4

ROI	GABA	Glx	tNAA	tCr
	2.5			
isthmus cingulate left	10.5 ± 3.0	8.2 ± 3.2	4.1 ± 3.0	5.0 ± 3.1
isthmus cingulate right	10.2 ± 2.8	8.1 ± 3.6	3.9 ± 2.9	4.9 ± 3.1
pallidum left	11.3 ± 3.9	7.6 ± 2.7	4.3 ± 2.4	5.3 ± 2.8
pallidum right	10.2 ± 3.3	8.2 ± 3.0	4.9 ± 2.6	5.7 ± 2.7
putamen left	11.1 ± 3.5	7.6 ± 2.7	4.5 ± 2.4	5.4 ± 2.7
putamen right	10.2 ± 2.9	8.1 ± 2.7	5.5 ± 2.9	5.8 ± 2.5
thalamus left	8.9 ± 2.9	7.3 ± 3.1	3.6 ± 2.7	4.6 ± 3.0
thalamus right	8.8 ± 2.8	7.3 ± 3.2	3.6 ± 2.6	4.5 ± 2.7

2. Supplement: Representative LCModel spectral fitting output.

The upper panel shows the *OFF spectrum*, while the lower panel shows the corresponding *difference spectrum* (ON–OFF). In each panel, the black line represents the measured spectrum, the red line the LCModel fit, and the residual is shown above the baseline.

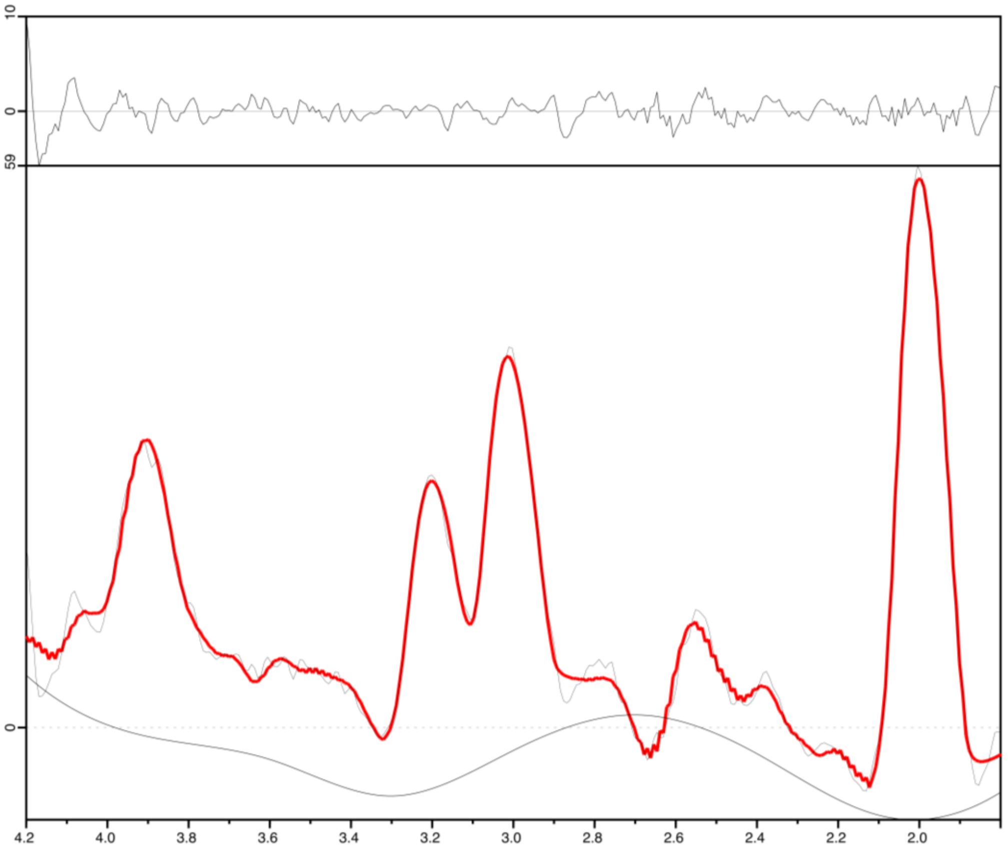

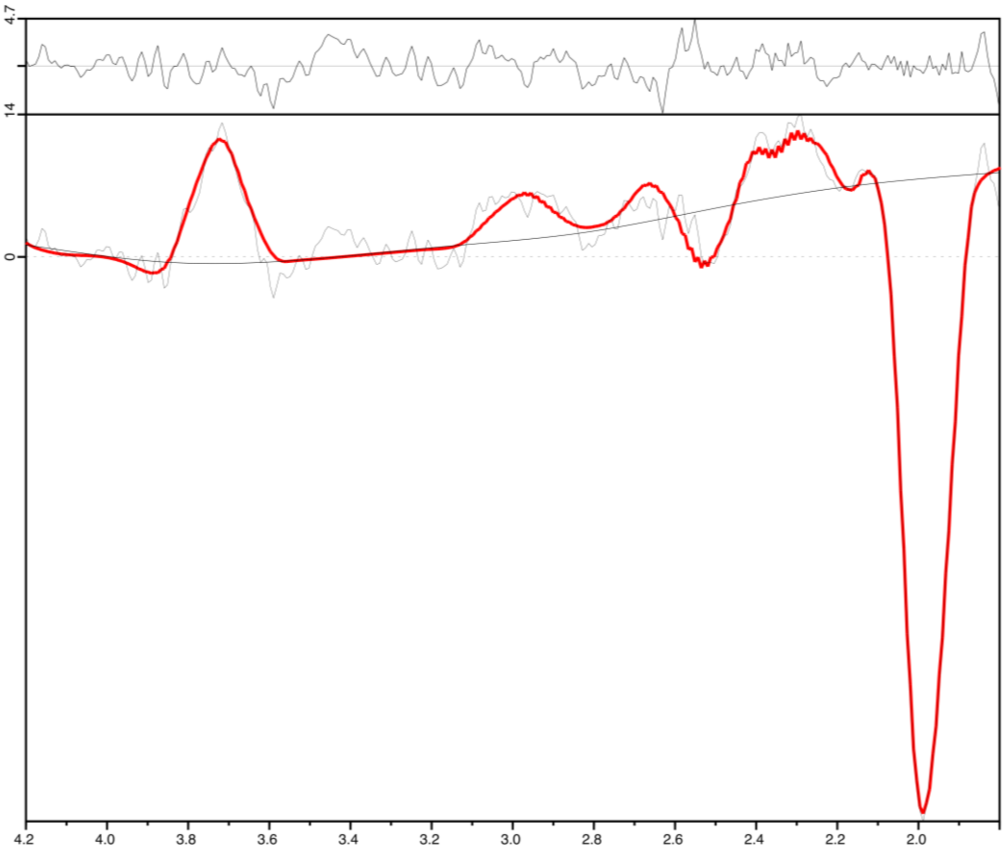

3. Supplement: Valid voxel counts and contributing subjects per ROI (ratio valid ROI only; mean ± SD [min–max] and % of total

Ratio: GABA/tCr

Ratio	ROI	N subjects	Numerator	Valid voxels mean ± SD [min–max]	% of total mean% ± SD% [min–max]	Denominator	Valid voxels mean ± SD [min–max]	% of total mean% ± SD% [min–max]
GABA/tCr	accumbens area left	58	GABA	342 ±87 [72–515]	98.2% ±3.7% [84.2–100.0]	tCr	342 ±85 [60–515]	100.0% ±0.0% [99.7–100.0]
GABA/tCr	accumbens area right	52	GABA	507 ±80 [233–707]	98.0% ±4.2% [81.7–100.0]	tCr	515 ±79 [231–707]	99.9% ±0.4% [97.4–100.0]
GABA/tCr	caudal anterior cingulate left	63	GABA	1810 ±547 [682–3131]	97.1% ±5.6% [80.1–100.0]	tCr	1891 ±546 [977–3131]	99.7% ±1.5% [88.4–100.0]
GABA/tCr	caudal anterior cingulate right	61	GABA	1880 ±477 [441–3045]	97.7% ±4.5% [83.4–100.0]	tCr	1942 ±395 [1221–3076]	99.3% ±2.8% [83.5–100.0]
GABA/tCr	caudate left	69	GABA	3300 ±560 [1415–4658]	97.3% ±5.1% [81.2–100.0]	tCr	3262 ±609 [1098–4574]	98.6% ±3.3% [82.8–100.0]
GABA/tCr	caudate right	66	GABA	3369 ±682 [770–4624]	97.4% ±5.0% [80.2–100.0]	tCr	3403 ±585 [1612–4533]	99.2% ±2.2% [88.4–100.0]
GABA/tCr	corpus callosum	72	GABA	2924 ±458 [1729–4115]	97.3% ±5.1% [81.2–100.0]	tCr	2917 ±441 [1526–4173]	98.0% ±4.5% [80.9–100.0]
GABA/tCr	hippocampus left	49	GABA	3882 ±419 [2884–4953]	97.0% ±5.4% [82.3–100.0]	tCr	3890 ±509 [1838–4665]	99.9% ±0.3% [97.7–100.0]

Ratio	ROI	N subjects	Numerator	Valid voxels mean ± SD [min–max]	% of total mean% ± SD% [min–max]	Denominator	Valid voxels mean ± SD [min–max]	% of total mean% ± SD% [min–max]
GABA/tCr	hippocampus right	49	GABA	3996 ±448 [2861–4847]	98.0% ±3.8% [84.7–100.0]	tCr	4059 ±461 [2620–4954]	99.8% ±0.8% [95.3–100.0]
GABA/tCr	insula left	66	GABA	6344 ±976 [3647–8620]	95.2% ±6.5% [80.7–100.0]	tCr	6506 ±988 [2745–8626]	99.6% ±1.8% [88.8–100.0]
GABA/tCr	insula right	62	GABA	6253 ±1058 [3040–8627]	96.6% ±4.9% [81.6–100.0]	tCr	6378 ±1005 [3034–8639]	99.8% ±0.7% [94.9–100.0]
GABA/tCr	isthmus cingulate left	71	GABA	2371 ±364 [1472–3085]	97.1% ±5.2% [80.6–100.0]	tCr	2423 ±359 [1586–3254]	99.2% ±2.5% [86.5–100.0]
GABA/tCr	isthmus cingulate right	70	GABA	2246 ±326 [1440–3065]	97.4% ±4.8% [82.9–100.0]	tCr	2287 ±328 [1652–3069]	99.3% ±2.9% [81.5–100.0]
GABA/tCr	pallidum left	71	GABA	2010 ±230 [871–2445]	98.8% ±3.4% [82.2–100.0]	tCr	1948 ±380 [286–2445]	99.9% ±0.9% [92.2–100.0]
GABA/tCr	pallidum right	71	GABA	1881 ±188 [1293–2375]	99.1% ±2.3% [88.1–100.0]	tCr	1841 ±269 [738–2375]	99.7% ±1.3% [92.0–100.0]
GABA/tCr	putamen left	74	GABA	4251 ±649 [1722–5579]	97.0% ±5.4% [80.2–100.0]	tCr	4219 ±753 [1289–5603]	99.6% ±2.3% [80.9–100.0]
GABA/tCr	putamen right	69	GABA	4393 ±601 [1983–5370]	96.6% ±5.5% [80.2–100.0]	tCr	4503 ±559 [2457–5393]	99.6% ±1.8% [88.6–100.0]
GABA/tCr	thalamus left	73	GABA	7755 ±823 [4408–9852]	99.0% ±2.9% [81.0–100.0]	tCr	7586 ±871 [4549–9344]	98.3% ±3.9% [80.3–100.0]
GABA/tCr	thalamus right	73	GABA	7434 ±905 [2584–9037]	98.0% ±4.6% [81.2–100.0]	tCr	7377 ±1077 [1505–9037]	98.7% ±3.7% [81.4–100.0]

Ratio: Glx/tCr

Ratio	ROI	N subjects	Numerator	Valid voxels mean ± SD [min–max]	% of total mean% ± SD% [min–max]	Denominator	Valid voxels mean ± SD [min–max]	% of total mean% ± SD% [min–max]
Glx/tCr	accumbens area left	81	Glx	362 ±98 [72–638]	99.4% ±2.5% [83.3–100.0]	tCr	351 ±95 [60–638]	100.0% ±0.1% [99.6–100.0]
Glx/tCr	accumbens area right	84	Glx	513 ±98 [60–716]	98.4% ±3.7% [81.1–100.0]	tCr	511 ±94 [41–707]	99.8% ±0.7% [94.2–100.0]
Glx/tCr	caudal anterior cingulate left	75	Glx	1835 ±489 [743–3131]	98.7% ±3.9% [81.3–100.0]	tCr	1842 ±504 [908–3131]	99.5% ±2.1% [87.1–100.0]
Glx/tCr	caudal anterior cingulate right	80	Glx	1932 ±556 [441–4395]	98.2% ±4.5% [80.9–100.0]	tCr	1911 ±494 [101–3394]	99.4% ±2.6% [83.5–100.0]
Glx/tCr	caudate left	78	Glx	3282 ±529 [1734–4492]	97.0% ±5.1% [80.5–100.0]	tCr	3200 ±667 [1098–4489]	98.7% ±3.1% [82.8–100.0]
Glx/tCr	caudate right	79	Glx	3366 ±677 [839–4533]	98.1% ±3.8% [82.3–100.0]	tCr	3372 ±671 [1053–4549]	99.3% ±2.0% [88.4–100.0]
Glx/tCr	corpus callosum	62	Glx	2765 ±430 [1730–4103]	95.4% ±6.5% [80.3–100.0]	tCr	2877 ±419 [1813–4173]	98.9% ±3.2% [84.6–100.0]
Glx/tCr	hippocampus left	84	Glx	3913 ±465 [2700–5167]	97.8% ±4.3% [82.2–100.0]	tCr	3896 ±527 [1838–5122]	99.3% ±2.0% [87.8–100.0]
Glx/tCr	hippocampus right	87	Glx	3999 ±498 [2226–5054]	97.9% ±4.2% [80.5–100.0]	tCr	4013 ±585 [1152–5054]	99.5% ±1.5% [90.7–100.0]
Glx/tCr	insula left	88	Glx	6569 ±924 [4121–8795]	98.7% ±3.4% [81.4–100.0]	tCr	6412 ±1036 [2745–8626]	99.7% ±1.5% [88.8–100.0]
Glx/tCr	insula right	90	Glx	6391 ±1083 [1401–8980]	98.1% ±4.2% [83.0–100.0]	tCr	6443 ±984 [3034–8639]	99.8% ±0.7% [94.9–100.0]

Ratio	ROI	N subjects	Numerator	Valid voxels mean ± SD [min–max]	% of total mean% ± SD% [min–max]	Denominator	Valid voxels mean ± SD [min–max]	% of total mean% ± SD% [min–max]
Glx/tCr	isthmus cingulate left	80	Glx	2304 ±362 [1423–2946]	96.5% ±5.3% [81.0–100.0]	tCr	2366 ±435 [974–3254]	99.5% ±1.9% [86.5–100.0]
Glx/tCr	isthmus cingulate right	81	Glx	2139 ±369 [982–2998]	95.9% ±6.1% [80.3–100.0]	tCr	2207 ±444 [285–3069]	99.4% ±2.7% [81.5–100.0]
Glx/tCr	pallidum left	85	Glx	2008 ±231 [871–2445]	98.8% ±3.1% [86.4–100.0]	tCr	1978 ±343 [286–2445]	99.9% ±0.8% [92.2–100.0]
Glx/tCr	pallidum right	83	Glx	1874 ±244 [504–2375]	99.0% ±3.0% [84.1–100.0]	tCr	1834 ±290 [704–2375]	99.8% ±1.2% [92.0–100.0]
Glx/tCr	putamen left	84	Glx	4402 ±521 [3057–5825]	98.7% ±3.3% [81.4–100.0]	tCr	4250 ±716 [1289–5603]	99.7% ±2.1% [80.9–100.0]
Glx/tCr	putamen right	83	Glx	4516 ±562 [2467–5888]	98.3% ±4.1% [82.2–100.0]	tCr	4536 ±632 [1813–5903]	99.7% ±1.4% [88.6–100.0]
Glx/tCr	thalamus left	74	Glx	7489 ±805 [4395–9344]	95.5% ±6.3% [80.3–100.0]	tCr	7607 ±926 [3208–9344]	98.5% ±3.7% [80.3–100.0]
Glx/tCr	thalamus right	76	Glx	7329 ±872 [2889–9028]	97.0% ±5.0% [81.0–100.0]	tCr	7350 ±1201 [1505–9037]	99.3% ±2.3% [85.6–100.0]

Ratio: tNAA/tCr

Ratio	ROI	N subjects	Numerator	Valid voxels mean ± SD [min–max]	% of total mean% ± SD% [min–max]	Denominator	Valid voxels mean ± SD [min–max]	% of total mean% ± SD% [min–max]
tNAA/tCr	accumbens area left	89	tNAA	353 ±90 [60–638]	99.9% ±0.4% [97.2–100.0]	tCr	353 ±90 [60–638]	100.0% ±0.1% [99.6–100.0]

Ratio	ROI	N subjects	Numerator	Valid voxels mean ± SD [min–max]	% of total mean% ± SD% [min–max]	Denominator	Valid voxels mean ± SD [min–max]	% of total mean% ± SD% [min–max]
tNAA/tCr	accumbens area right	92	tNAA	511 ±91 [41–707]	99.4% ±2.5% [81.7–100.0]	tCr	513 ±93 [41–707]	99.9% ±0.7% [94.2–100.0]
tNAA/tCr	caudal anterior cingulate left	85	tNAA	1834 ±523 [973–3131]	99.1% ±3.1% [83.6–100.0]	tCr	1843 ±521 [908–3131]	99.6% ±2.0% [87.1–100.0]
tNAA/tCr	caudal anterior cingulate right	86	tNAA	1941 ±435 [877–3138]	99.0% ±3.3% [81.2–100.0]	tCr	1945 ±438 [877–3394]	99.2% ±2.9% [83.5–100.0]
tNAA/tCr	caudate left	83	tNAA	3245 ±628 [1105–4477]	98.4% ±3.1% [87.9–100.0]	tCr	3261 ±626 [1226–4574]	99.0% ±2.8% [82.8–100.0]
tNAA/tCr	caudate right	89	tNAA	3291 ±697 [873–4549]	97.9% ±4.2% [82.9–100.0]	tCr	3331 ±686 [1053–4549]	99.2% ±2.3% [88.3–100.0]
tNAA/tCr	corpus callosum	73	tNAA	2863 ±462 [1529–4173]	96.8% ±5.5% [80.1–100.0]	tCr	2917 ±481 [1526–4173]	98.5% ±4.2% [80.9–100.0]
tNAA/tCr	hippocampus left	93	tNAA	3862 ±590 [1709–5122]	99.3% ±2.1% [87.8–100.0]	tCr	3865 ±593 [1584–5122]	99.3% ±1.9% [87.8–100.0]
tNAA/tCr	hippocampus right	95	tNAA	3991 ±578 [1165–5054]	99.3% ±2.0% [87.7–100.0]	tCr	3995 ±571 [1152–5054]	99.5% ±1.5% [90.7–100.0]
tNAA/tCr	insula left	95	tNAA	6405 ±1048 [2744–8626]	99.4% ±2.2% [87.0–100.0]	tCr	6419 ±1033 [2745–8626]	99.6% ±1.6% [88.8–100.0]
tNAA/tCr	insula right	94	tNAA	6364 ±1034 [2918–8639]	99.3% ±2.3% [86.3–100.0]	tCr	6392 ±1035 [3034–8639]	99.8% ±0.7% [94.9–100.0]
tNAA/tCr	isthmus cingulate left	88	tNAA	2339 ±432 [567–3254]	98.1% ±4.2% [82.8–100.0]	tCr	2372 ±441 [529–3254]	99.4% ±2.0% [86.5–100.0]

Ratio	ROI	N subjects	Numerator	Valid voxels mean ± SD [min–max]	% of total mean% ± SD% [min–max]	Denominator	Valid voxels mean ± SD [min–max]	% of total mean% ± SD% [min–max]
tNAA/tCr	isthmus cingulate right	85	tNAA	2190 ±424 [234–2994]	98.0% ±4.3% [81.4–100.0]	tCr	2224 ±437 [285–3069]	99.6% ±1.9% [87.7–100.0]
tNAA/tCr	pallidum left	91	tNAA	1959 ±347 [285–2445]	99.5% ±2.0% [86.6–100.0]	tCr	1967 ±345 [286–2445]	99.9% ±0.8% [92.2–100.0]
tNAA/tCr	pallidum right	89	tNAA	1813 ±301 [704–2375]	99.5% ±2.1% [83.2–100.0]	tCr	1820 ±301 [704–2375]	99.9% ±0.8% [92.8–100.0]
tNAA/tCr	putamen left	92	tNAA	4213 ±726 [1290–5578]	99.4% ±2.0% [88.0–100.0]	tCr	4222 ±726 [1289–5603]	99.6% ±2.1% [80.9–100.0]
tNAA/tCr	putamen right	91	tNAA	4472 ±620 [1813–5821]	99.2% ±3.0% [82.6–100.0]	tCr	4489 ±642 [1813–5903]	99.5% ±2.1% [83.6–100.0]
tNAA/tCr	thalamus left	78	tNAA	7617 ±786 [4510–9344]	98.3% ±4.1% [81.1–100.0]	tCr	7671 ±796 [4549–9344]	98.9% ±3.1% [80.3–100.0]
tNAA/tCr	thalamus right	82	tNAA	7177 ±1196 [1307–9037]	97.0% ±5.5% [82.4–100.0]	tCr	7319 ±1177 [1505–9037]	99.0% ±3.0% [85.3–100.0]

Ratio: GABA/tNAA

Ratio	ROI	N subjects	Numerator	Valid voxels mean ± SD [min–max]	% of total mean% ± SD% [min–max]	Denominator	Valid voxels mean ± SD [min–max]	% of total mean% ± SD% [min–max]
GABA/tNAA	accumbens area left	57	GABA	341 ±87 [72–515]	98.3% ±3.5% [84.2–100.0]	tNAA	345 ±83 [60–515]	99.9% ±0.5% [97.2–100.0]
GABA/tNAA	accumbens area right	52	GABA	507 ±80 [233–707]	98.0% ±4.2% [81.7–100.0]	tNAA	514 ±79 [231–707]	99.8% ±1.1% [92.5–100.0]

Ratio	ROI	N subjects	Numerator	Valid voxels mean ± SD [min–max]	% of total mean% ± SD% [min–max]	Denominator	Valid voxels mean ± SD [min–max]	% of total mean% ± SD% [min–max]
GABA/tNAA	caudal anterior cingulate left	64	GABA	1795 ±554 [682–3131]	97.1% ±5.5% [80.1–100.0]	tNAA	1874 ±547 [982–3131]	98.9% ±3.4% [83.6–100.0]
GABA/tNAA	caudal anterior cingulate right	59	GABA	1888 ±482 [441–3045]	97.8% ±4.4% [83.4–100.0]	tNAA	1946 ±399 [1221–3076]	99.1% ±3.3% [81.2–100.0]
GABA/tNAA	caudate left	67	GABA	3291 ±565 [1415–4658]	97.5% ±4.7% [81.5–100.0]	tNAA	3266 ±558 [1745–4477]	98.2% ±3.1% [87.6–100.0]
GABA/tNAA	caudate right	67	GABA	3381 ±684 [770–4624]	97.3% ±5.0% [80.2–100.0]	tNAA	3344 ±596 [1696–4533]	97.7% ±4.4% [82.1–100.0]
GABA/tNAA	corpus callosum	64	GABA	2924 ±464 [1729–4115]	97.1% ±5.3% [81.2–100.0]	tNAA	2890 ±416 [1860–4173]	96.7% ±5.6% [80.1–100.0]
GABA/tNAA	hippocampus left	49	GABA	3882 ±419 [2884–4953]	97.0% ±5.4% [82.3–100.0]	tNAA	3881 ±524 [1838–4674]	99.7% ±1.4% [91.9–100.0]
GABA/tNAA	hippocampus right	49	GABA	3996 ±448 [2861–4847]	98.0% ±3.8% [84.7–100.0]	tNAA	4053 ±467 [2620–4954]	99.6% ±1.1% [94.9–100.0]
GABA/tNAA	insula left	66	GABA	6344 ±976 [3647–8620]	95.2% ±6.5% [80.7–100.0]	tNAA	6508 ±1003 [2744–8626]	99.6% ±1.9% [88.6–100.0]
GABA/tNAA	insula right	62	GABA	6253 ±1058 [3040–8627]	96.6% ±4.9% [81.6–100.0]	tNAA	6361 ±1000 [3037–8639]	99.6% ±1.9% [88.3–100.0]
GABA/tNAA	isthmus cingulate left	71	GABA	2376 ±362 [1472–3085]	97.2% ±5.0% [80.6–100.0]	tNAA	2389 ±350 [1500–3254]	97.7% ±4.4% [82.8–100.0]
GABA/tNAA	isthmus cingulate right	69	GABA	2252 ±327 [1440–3065]	97.3% ±4.9% [82.9–100.0]	tNAA	2251 ±307 [1652–2994]	97.6% ±4.7% [81.4–100.0]

Ratio	ROI	N subjects	Numerator	Valid voxels mean ± SD [min–max]	% of total mean% ± SD% [min–max]	Denominator	Valid voxels mean ± SD [min–max]	% of total mean% ± SD% [min–max]
GABA/tNAA	pallidum left	71	GABA	2010 ±230 [871–2445]	98.8% ±3.4% [82.2–100.0]	tNAA	1941 ±382 [285–2445]	99.5% ±2.0% [86.6–100.0]
GABA/tNAA	pallidum right	70	GABA	1875 ±189 [1293–2375]	99.1% ±2.3% [88.1–100.0]	tNAA	1832 ±271 [738–2375]	99.7% ±1.3% [92.1–100.0]
GABA/tNAA	putamen left	74	GABA	4251 ±649 [1722–5579]	97.0% ±5.4% [80.2–100.0]	tNAA	4212 ±753 [1290–5578]	99.4% ±1.9% [88.0–100.0]
GABA/tNAA	putamen right	69	GABA	4393 ±602 [1983–5370]	96.8% ±5.3% [80.2–100.0]	tNAA	4485 ±567 [2457–5379]	99.4% ±2.3% [86.8–100.0]
GABA/tNAA	thalamus left	65	GABA	7739 ±823 [4408–9344]	98.9% ±3.2% [81.0–100.0]	tNAA	7579 ±809 [4510–9344]	97.9% ±4.6% [81.1–100.0]
GABA/tNAA	thalamus right	67	GABA	7449 ±930 [2584–9037]	98.1% ±4.3% [81.6–100.0]	tNAA	7242 ±1108 [1307–9037]	96.9% ±5.5% [82.4–100.0]

Ratio: Glx/tNAA

Ratio	ROI	N subjects	Numerator	Valid voxels mean ± SD [min–max]	% of total mean% ± SD% [min–max]	Denominator	Valid voxels mean ± SD [min–max]	% of total mean% ± SD% [min–max]
Glx/tNAA	accumbens area left	80	Glx	362 ±98 [72–638]	99.4% ±2.5% [83.3–100.0]	tNAA	354 ±93 [60–638]	99.7% ±2.2% [80.5–100.0]
Glx/tNAA	accumbens area right	83	Glx	513 ±99 [60–716]	98.4% ±3.7% [81.1–100.0]	tNAA	510 ±92 [41–707]	99.4% ±2.6% [81.7–100.0]
Glx/tNAA	caudal anterior cingulate left	76	Glx	1826 ±496 [743–3131]	98.6% ±3.9% [81.3–100.0]	tNAA	1844 ±491 [973–3131]	99.3% ±2.6% [83.6–100.0]

Ratio	ROI	N subjects	Numerator	Valid voxels mean ± SD [min–max]	% of total mean% ± SD% [min–max]	Denominator	Valid voxels mean ± SD [min–max]	% of total mean% ± SD% [min–max]
Glx/tNAA	caudal anterior cingulate right	77	Glx	1933 ±566 [441–4395]	98.2% ±4.5% [80.9–100.0]	tNAA	1935 ±452 [877–3138]	99.2% ±3.1% [81.2–100.0]
Glx/tNAA	caudate left	76	Glx	3254 ±527 [1734–4492]	96.8% ±5.2% [80.5–100.0]	tNAA	3233 ±593 [1105–4477]	98.4% ±3.0% [87.6–100.0]
Glx/tNAA	caudate right	77	Glx	3350 ±673 [839–4533]	98.1% ±3.9% [82.3–100.0]	tNAA	3311 ±674 [873–4549]	97.8% ±4.2% [82.9–100.0]
Glx/tNAA	corpus callosum	59	Glx	2801 ±425 [1730–4103]	96.1% ±5.8% [81.7–100.0]	tNAA	2851 ±426 [1529–4173]	97.7% ±4.8% [80.1–100.0]
Glx/tNAA	hippocampus left	83	Glx	3925 ±456 [2700–5167]	97.9% ±4.0% [82.2–100.0]	tNAA	3907 ±513 [1838–5122]	99.2% ±2.2% [87.8–100.0]
Glx/tNAA	hippocampus right	87	Glx	3999 ±498 [2226–5054]	97.9% ±4.2% [80.5–100.0]	tNAA	4008 ±591 [1165–5054]	99.3% ±2.1% [87.7–100.0]
Glx/tNAA	insula left	88	Glx	6569 ±924 [4121–8795]	98.7% ±3.4% [81.4–100.0]	tNAA	6389 ±1050 [2744–8626]	99.3% ±2.3% [87.0–100.0]
Glx/tNAA	insula right	90	Glx	6391 ±1083 [1401–8980]	98.1% ±4.2% [83.0–100.0]	tNAA	6417 ±969 [3037–8639]	99.5% ±1.9% [88.3–100.0]
Glx/tNAA	isthmus cingulate left	80	Glx	2309 ±363 [1423–2946]	96.5% ±5.3% [81.0–100.0]	tNAA	2358 ±402 [1016–3254]	98.3% ±3.9% [82.8–100.0]
Glx/tNAA	isthmus cingulate right	81	Glx	2146 ±372 [982–2998]	96.0% ±5.9% [80.3–100.0]	tNAA	2181 ±432 [234–2994]	98.1% ±4.3% [81.4–100.0]
Glx/tNAA	pallidum left	84	Glx	2006 ±232 [871–2445]	98.8% ±3.1% [86.4–100.0]	tNAA	1970 ±347 [285–2445]	99.4% ±2.1% [86.6–100.0]

Ratio	ROI	N subjects	Numerator	Valid voxels mean ± SD [min–max]	% of total mean% ± SD% [min–max]	Denominator	Valid voxels mean ± SD [min–max]	% of total mean% ± SD% [min–max]
Glx/tNAA	pallidum right	80	Glx	1870 ±250 [504–2375]	99.3% ±2.6% [84.1–100.0]	tNAA	1821 ±296 [704–2375]	99.5% ±2.2% [83.2–100.0]
Glx/tNAA	putamen left	83	Glx	4398 ±522 [3057–5825]	98.7% ±3.4% [81.4–100.0]	tNAA	4246 ±715 [1290–5578]	99.5% ±1.7% [89.0–100.0]
Glx/tNAA	putamen right	82	Glx	4521 ±562 [2467–5888]	98.6% ±3.7% [82.2–100.0]	tNAA	4503 ±629 [1813–5821]	99.2% ±2.8% [82.6–100.0]
Glx/tNAA	thalamus left	66	Glx	7521 ±810 [4395–9344]	95.8% ±6.1% [80.9–100.0]	tNAA	7685 ±692 [6397–9344]	98.3% ±4.2% [81.1–100.0]
Glx/tNAA	thalamus right	70	Glx	7365 ±868 [2889–9028]	97.0% ±4.9% [81.0–100.0]	tNAA	7275 ±1176 [1307–9037]	97.4% ±5.1% [82.4–100.0]

Ratio: tCr/tNAA

Ratio	ROI	N subjects	Numerator	Valid voxels mean ± SD [min–max]	% of total mean% ± SD% [min–max]	Denominator	Valid voxels mean ± SD [min–max]	% of total mean% ± SD% [min–max]
tCr/tNAA	accumbens area left	89	tCr	353 ±90 [60–638]	100.0% ±0.1% [99.6–100.0]	tNAA	353 ±90 [60–638]	99.9% ±0.4% [97.2–100.0]
tCr/tNAA	accumbens area right	92	tCr	513 ±93 [41–707]	99.9% ±0.7% [94.2–100.0]	tNAA	511 ±91 [41–707]	99.4% ±2.5% [81.7–100.0]
tCr/tNAA	caudal anterior cingulate left	85	tCr	1843 ±521 [908–3131]	99.6% ±2.0% [87.1–100.0]	tNAA	1834 ±523 [973–3131]	99.1% ±3.1% [83.6–100.0]
tCr/tNAA	caudal anterior cingulate right	86	tCr	1945 ±438 [877–3394]	99.2% ±2.9% [83.5–100.0]	tNAA	1941 ±435 [877–3138]	99.0% ±3.3% [81.2–100.0]

Ratio	ROI	N subjects	Numerator	Valid voxels mean ± SD [min–max]	% of total mean% ± SD% [min–max]	Denominator	Valid voxels mean ± SD [min–max]	% of total mean% ± SD% [min–max]
tCr/tNAA	caudate left	83	tCr	3261 ±626 [1226–4574]	99.0% ±2.8% [82.8–100.0]	tNAA	3245 ±628 [1105–4477]	98.4% ±3.1% [87.9–100.0]
tCr/tNAA	caudate right	89	tCr	3331 ±686 [1053–4549]	99.2% ±2.3% [88.3–100.0]	tNAA	3291 ±697 [873–4549]	97.9% ±4.2% [82.9–100.0]
tCr/tNAA	corpus callosum	73	tCr	2917 ±481 [1526–4173]	98.5% ±4.2% [80.9–100.0]	tNAA	2863 ±462 [1529–4173]	96.8% ±5.5% [80.1–100.0]
tCr/tNAA	hippocampus left	93	tCr	3865 ±593 [1584–5122]	99.3% ±1.9% [87.8–100.0]	tNAA	3862 ±590 [1709–5122]	99.3% ±2.1% [87.8–100.0]
tCr/tNAA	hippocampus right	95	tCr	3995 ±571 [1152–5054]	99.5% ±1.5% [90.7–100.0]	tNAA	3991 ±578 [1165–5054]	99.3% ±2.0% [87.7–100.0]
tCr/tNAA	insula left	95	tCr	6419 ±1033 [2745–8626]	99.6% ±1.6% [88.8–100.0]	tNAA	6405 ±1048 [2744–8626]	99.4% ±2.2% [87.0–100.0]
tCr/tNAA	insula right	94	tCr	6392 ±1035 [3034–8639]	99.8% ±0.7% [94.9–100.0]	tNAA	6364 ±1034 [2918–8639]	99.3% ±2.3% [86.3–100.0]
tCr/tNAA	isthmus cingulate left	88	tCr	2372 ±441 [529–3254]	99.4% ±2.0% [86.5–100.0]	tNAA	2339 ±432 [567–3254]	98.1% ±4.2% [82.8–100.0]
tCr/tNAA	isthmus cingulate right	85	tCr	2224 ±437 [285–3069]	99.6% ±1.9% [87.7–100.0]	tNAA	2190 ±424 [234–2994]	98.0% ±4.3% [81.4–100.0]
tCr/tNAA	pallidum left	91	tCr	1967 ±345 [286–2445]	99.9% ±0.8% [92.2–100.0]	tNAA	1959 ±347 [285–2445]	99.5% ±2.0% [86.6–100.0]
tCr/tNAA	pallidum right	89	tCr	1820 ±301 [704–2375]	99.9% ±0.8% [92.8–100.0]	tNAA	1813 ±301 [704–2375]	99.5% ±2.1% [83.2–100.0]

Ratio	ROI	N subjects	Numerator	Valid voxels mean ± SD [min–max]	% of total mean% ± SD% [min–max]	Denominator	Valid voxels mean ± SD [min–max]	% of total mean% ± SD% [min–max]
tCr/tNAA	putamen left	92	tCr	4222 ±726 [1289–5603]	99.6% ±2.1% [80.9–100.0]	tNAA	4213 ±726 [1290–5578]	99.4% ±2.0% [88.0–100.0]
tCr/tNAA	putamen right	91	tCr	4489 ±642 [1813–5903]	99.5% ±2.1% [83.6–100.0]	tNAA	4472 ±620 [1813–5821]	99.2% ±3.0% [82.6–100.0]
tCr/tNAA	thalamus left	78	tCr	7671 ±796 [4549–9344]	98.9% ±3.1% [80.3–100.0]	tNAA	7617 ±786 [4510–9344]	98.3% ±4.1% [81.1–100.0]
tCr/tNAA	thalamus right	82	tCr	7319 ±1177 [1505–9037]	99.0% ±3.0% [85.3–100.0]	tNAA	7177 ±1196 [1307–9037]	97.0% ±5.5% [82.4–100.0]

4. Supplement: Detailed results of continuous predictor models

Association of metabolite ratios with EEG IRDA/IRTA in BPD patients

In the ROI-by-predictor mixed-effects model, higher IRDA/IRTA per minute before HV was significantly related to Glx/tCr independent of ROI [F(1, 54.77) = 6.24, p = 0.016, p_{BHw} = 0.062, p_{BHg} = 0.373]. Similarly, IRDA/IRTA per minute before HV was associated with Glx/tNAA [F(1, 52.50) = 4.65, p = 0.036, p_{BHw} = 0.143, p_{BHg} = 0.428]. In follow-up ROI-wise comparisons using emmeans, positive associations between Glx/tCr and IRDA/IRTA per minute before HV in the left accumbens area (β = 0.055, p < 0.001), right accumbens area (β = 0.043, p = 0.002), and right caudal anterior cingulate (β = 0.033, p = 0.022) have been revealed. Similarly, Glx/tNAA was positively associated with IRDA/IRTA per minute before HV in the left accumbens area (β = 0.059, p = 0.002) and right accumbens area (β = 0.062, p < 0.001). The IRDA difference score was not significantly associated with the neurometabolite ratios, and no significant IRDA difference × ROI interaction was observed.

Association of metabolite ratios with psychometry in BPD patients

In ROI-by-predictor mixed-effects model, the mean BSL-supplement score was significantly associated with tCr/tNAA [F(1, 138.31) = 4.30, p = 0.040, p_{BHw} = 0.279, p_{BHg} = 0.902]. Further, a significant ROI × BSL-supplement interaction for tCr/tNAA was found [F(18, 1037.25) = 2.32, p = 0.001, p_{BHw} = 0.019, p_{BHg} = 0.115]. In post-hoc robust analyses for each ROI, the mean BSL-supplement score was positively associated with tCr/tNAA in the left caudate (β = 0.179, p = 0.018), right caudate (β = 0.369, p < 0.001), right pallidum (β = 0.191, p = 0.011) and right putamen (β = 0.18, p = 0.016). For all other psychometric scores, no significant association with the neurometabolite ratios, and no significant interaction with ROI was observed.

Association of metabolite ratios with neuropsychological testing in BPD patients

In ROI-by-predictor mixed-effects models for GABA/tCr, alertness (warning tone) showed a significant main effect [F(1, 52.30) = 4.20, p = 0.046, p_{BHw} = 0.273, p_{BHg} = 0.348]. Significant predictor × ROI interactions were observed for IQ (CFT-20 R) [F(18, 654.81) = 2.22, p = 0.003, p_{BHw} = 0.032, p_{BHg} = 0.056], VLMT learning [F(18, 712.42) = 2.44, p = 0.001, p_{BHw} = 0.018, p_{BHg} = 0.027] and VLMT recognition [F(18, 712.17) = 1.76, p = 0.027, p_{BHw} = 0.215, p_{BHg} = 0.290]. Post-hoc associations across individual ROIs demonstrated that reduced alertness performance (warning tone)

was significantly associated with higher GABA/tCr levels in the left caudate (β = -0.002, p = 0.025), left pallidum (β = -0.003, p = 0.009), and left putamen (β = -0.003, p = 0.003). Higher GABA/tCr was also significantly associated with superior IQ performance (CFT-20 R) in the left caudate (β = 0.012, p < 0.001), poorer VLMT learning performance in the left caudate (β = -0.02, p < 0.001), and better VLMT recognition in the left caudate (β = 0.08, p = 0.003).

In ROI × predictor mixed-effects models for Glx/tCr**,** divided attention omissions and working memory errors showed significant predictor × ROI interactions**,** indicating that the strength of the association differed across ROIs (divided attention omissions: $F(18, 855.80) = 1.64$, p = 0.046, p_{BHw} = 0.348, p_{BHg} = 0.348; working memory errors: $F(18, 853.80) = 1.97$, p = 0.009, p_{BHw} = 0.223, p_{BHg} = 0.149). In post-hoc ROI-wise analyses, more divided attention omissions were associated with higher Glx/tCr in the left caudal anterior cingulate (β = 0.103, p = 0.012), right hippocampus (β = 0.094, p = 0.019), and right isthmus cingulate (β = 0.077, p = 0.045). More working-memory errors were associated with higher Glx/tCr in the left caudal anterior cingulate (β = 0.041, p < 0.001), right caudal anterior cingulate (β = 0.035, p = 0.004), and right isthmus cingulate (β = 0.025, p = 0.039).

For Glx/tNAA, ROI × predictor mixed-effects models indicated significant alertness × ROI interactions [no warning tone: F(18, 833.51) = 3.54, p < 0.001, p_{BHw} < 0.001, p_{BHg} < 0.001; warning tone: F(18, 834.16) = 3.32, p < 0.001, p_{BHw} < 0.001, p_{BHg} < 0.001]. The corresponding main effects for alertness were significant [no warning tone: F(1, 51.93) = 5.10, p = 0.028, p_{BHw} = 0.169, p_{BHg} = 0.29; warning tone: F(1, 51.41) = 5.80, p = 0.020, p_{BHw} = 0.157, p_{BHg} = 0.258]. In post-hoc ROI-wise analyses, higher alertness scores were associated with higher Glx/tNAA (no warning tone) in the left caudal anterior cingulate (β = 0.006, p = 0.015) and right thalamus (β = 0.025, p < 0.001). Similarly, higher alertness scores (warning tone) were associated with higher Glx/tNAA in the left caudal anterior cingulate (β = 0.007, p = 0.027) and right thalamus (β = 0.029, p < 0.001).

For tCr/tNAA, alertness (no warning tone) showed both an overall association (main effect) and ROI-dependent effects [main effect: F(18, 959.54) = 1.69, p = 0.035, p_{BHw} = 0.17, p_{BHg} = 0.313; interaction: F(1, 62.32) = 6.62, p = 0.012, p_{BHw} = 0.149, p_{BHg} = 0.179]. ROI-dependent effects were also observed for alertness with warning tone [F(18, 959.44) = 1.78, p = 0.023, p_{BHw} = 0.17, p_{BHg} = 0.281] and for phasic alertness [F(18, 958.24) = 2.04, p = 0.006, p_{BHw} = 0.149, p_{BHg} = 0.116]. In post-hoc robust ROI-wise analyses, higher tCr/tNAA was associated with better alertness performance (no warning tone) in the left caudate (β = 0.003, p = 0.043), right caudate (β = 0.004, p = 0.003), left pallidum (β = 0.004, p = 0.040), right pallidum (β = 0.003, p = 0.023), right putamen (β = 0.003, p = 0.013), and right thalamus (β = 0.007, p < 0.001). Higher tCr/tNAA was also associated with better alertness performance (warning tone) in the left pallidum (β = 0.005, p = 0.020), right putamen (β = 0.004, p = 0.019), and right thalamus (β = 0.007,

$p < 0.001$). Finally, higher tCr/tNAA was associated with better phasic alertness in the left accumbens area ($\beta = 4.357$, $p < 0.001$), left caudate ($\beta = 2.158$, $p = 0.017$), and right caudate ($\beta = 2.488$, $p = 0.006$).

For tNAA/tCr, mixed-effects models showed significant predictor × ROI interactions for alertness [no warning tone: $F(18, 958.08) = 2.32$, $p = 0.001$, $p_{BHw} = 0.012$, $p_{BHg} = 0.035$; warning tone: $F(18, 958.23) = 3.29$, $p < 0.001$, $p_{BHw} < 0.001$, $p_{BHg} < 0.001$], for VLMT learning ($F(18, 1034.27) = 2.31$, $p = 0.001$, $p_{BHw} = 0.012$, $p_{BHg} = 0.035$), and IQ (CFT-20 R) [$F(18, 902.42) = 1.68$, $p = 0.037$, $p_{BHw} = 0.221$, $p_{BHg} = 0.313$). In ROI-specific follow-up analyses, higher tNAA/tCr was associated with poorer alertness (no warning tone) in the left pallidum ($\beta = -0.004$, $p = 0.038$), right pallidum ($\beta = -0.003$, $p = 0.017$), and right putamen ($\beta = -0.004$, $p = 0.002$), and with poorer alertness (warning tone) in the left pallidum ($\beta = -0.004$, $p = 0.023$), right pallidum ($\beta = -0.004$, $p = 0.034$), left putamen ($\beta = -0.004$, $p = 0.042$), and right putamen ($\beta = -0.006$, $p < 0.001$). Higher tNAA/tCr was also associated with poorer IQ performance in the left caudal anterior cingulate ($\beta = -0.015$, $p = 0.003$) and poorer VLMT learning performance in the left caudate ($\beta = -0.023$, $p = 0.005$) and right caudal anterior cingulate ($\beta = -0.019$, $p = 0.020$).
